# Chemotherapy-related cognitive impairment and non-pharmacological interventions targeting the nervous system: a systematic review

**DOI:** 10.3389/fpsyt.2026.1789794

**Published:** 2026-06-02

**Authors:** Norbert Dosa, Intouch Limvaree, Camila Bonin Pinto, Faddi Saleh Velez, Zalan Kaposzta, Zsofia Szarvas, Zachary C. Pope, Anna Csiszar, Wajeeha Razaq, Ryan D. Nipp, Andriy Yabluchanskiy, Peter Mukli

**Affiliations:** 1 Department of Neurosurgery, University of Oklahoma Health Campus, Oklahoma City, OK, United States; 2 Oklahoma Center for Geroscience and Healthy Brain Aging, University of Oklahoma Health Campus, Oklahoma City, OK, United States; 3 International Training Program in Geroscience, Doctoral College, Health Sciences Divisions/Institute of Preventive Medicine and Public Health, Semmelweis University, Budapest, Hungary; 4 Institute of Preventive Medicine and Public Health, Semmelweis University, Budapest, Hungary; 5 Brain Stimulation and Neurorehabilitation Laboratory, Department of Neurology, University of Oklahoma Health Campus, Oklahoma City, OK, United States; 6 Brain Stimulation and Neurorehabilitation Laboratory, Department of Neurosurgery, University of Oklahoma Health Campus, Oklahoma City, OK, United States; 7 Molecular Medicine, Department of Physiology, Semmelweis University, Budapest, Hungary; 8 Department of Health Promotion Sciences, Hudson College of Public Health, University of Oklahoma Health Campus, Oklahoma City, OK, United States; 9 TSET Health Promotion Research Center, Stephenson Cancer Center, University of Oklahoma Health Campus, Oklahoma City, OK, United States; 10 OU Health Stephenson Cancer Center, University of Oklahoma Health Sciences, Oklahoma City, OK, United States

**Keywords:** acupuncture, brain stimulation, chemobrain, chemotherapy-induced cognitive impairment, chemotherapy-related cognitive dysfunction, chemotherapy-related cognitive impairment, cognitive rehabilitation

## Abstract

**Background:**

Chemotherapy-related cognitive impairment (CRCI) represents an increasingly recognized problem in the growing cancer survivor population within the US and worldwide. CRCI is characterized by deficits in memory, sustained attention, and executive function, which significantly worsens the cancer survivors’ quality of life. An increasing number of studies have been conducted using novel intervention approaches aimed at mitigating CRCI. In this systematic review, we sought to summarize the current evidence of cognitive improvement in cancer survivors receiving non-pharmacological interventions with neurostimulatory effects following chemotherapy.

**Methods:**

We screened five databases (Embase, MEDLINE, PubMED, Scopus, and PsycINFO) for original articles reporting the administration of any type of brain stimulation or complementary/alternative therapies targeting the central and/or peripheral nervous system to improve cognitive outcomes in cancer survivors reporting CRCI. We systematically extracted information from each eligible study using participant, intervention, comparison, outcome(s), and study design (PICOS) framework according to Cochrane recommendations. We used the critical appraisal tool by Joanna Briggs Institute to assess the risk of bias.

**Results:**

After screening 2,708 manuscripts, we performed a full-text review of 77 papers and identified 17 studies that met our inclusion criteria: nine randomized controlled trials, four case reports, one case series, two quasi-experimental study, and one cohort study. We identified seven studies which focused on CRCI and 10 others where cognitive function was properly reported for inclusion. Subjective and objective cognitive outcome measures reflecting overall performance, attention, working memory, processing speed, and quality of life with separate cognitive function reporting were assessed in patient(s) treated with transcranial direct current stimulation, transcranial magnetic stimulation, multisensory stimulation, Flexyx neurotherapy system, acupuncture, and electroacupuncture. Mild improvements in some of the cognitive outcome measures were observed in all studies. The weaknesses of these studies can be attributed to insufficient statistical power and testing, lack of a control group, randomization, blinding, and incorrect statistical methods.

**Discussion and conclusion:**

We found only a handful of trials reporting cognitive outcomes in CRCI interventions, with small sample sizes and biased study designs limiting the validity of the statistically significant findings. Our systematic review provides rationale for assessing the impact of non-pharmacological neurostimulatory techniques on CRCI in large-scale randomized controlled trials.

## 1 Introduction

In 2022, an estimated 2 million new cancer cases were reported globally (1). In the US, more than 18 million individuals are living with a history of cancer, with this figure expected to rise to over 22 million by 2035 (2). Advances in prevention along with continued improvements in early detection and treatment have contributed to significant gains in survival rates, rising from under 50% in the 1980s to close to 70% by the late 2000s (3). Chemotherapy has contributed to these improved survival rates. However, despite its therapeutic benefits, chemotherapy is often accompanied by notable side effects, with cognitive issues among the side effects most negatively impacting the cancer survivors’ quality of life (4, 5). Often referred to as chemotherapy-related cognitive impairment (CRCI) (a.k.a., chemotherapy-induced cognitive impairment or “chemo-brain”) (6–8), CRCI affects the objective and subjective measurements of memory, executive function, attention, and processing speed (9–12). As CRCI can persist long after chemotherapy treatment ends (13–16), research continues to be conducted on non-pharmacological interventions capable of improving cognitive function and, thus, the quality of life among cancer survivors.

Preclinical studies of CRCI have helped to elucidate the underlying mechanisms of CRCI, thereby informing potential interventions. These studies suggest that commonly administered chemotherapy agents (e.g., cisplatin, methotrexate, doxorubicin, and paclitaxel) may promote CRCI, potentially through the senescence of cerebrovascular endothelium (17) as well as blood–brain barrier disruption and the ensuing neuroinflammation (18–22). Accumulating data from clinical studies have characterized chemotherapy-induced brain damage; structural brain imaging has revealed reduced thickness in the prefrontal cortex and hippocampal atrophy in cancer survivors experiencing cognitive deficits following chemotherapy (23–25). Functional brain imaging studies have also linked CRCI to decreased neural activity in regions associated with memory processes and impaired functional connectivity, disrupting resting-state networks underlying normal brain functions (23, 26–30).

Importantly, no pharmacological trial to date has demonstrated clinically significant improvement in cognitive function in patients with CRCI, with pharmacological agents having their own side effects. Most non-pharmacological, non-invasive interventions have a lower side effect profile than pharmacological agents, making these approaches attractive for mitigating CRCI (31). Prior work has shown that lifestyle interventions, including regular aerobic exercise training and adherence to time-restricted eating or other dietary programs, may help to improve cognitive functioning in older adults (32–34) and high-risk patient populations (35), including breast cancer survivors (36). However, maintenance of these health behaviors is often challenging in clinical trials and real-world scenarios. Increasingly, evidence suggests that cognitive training, which harnesses the mechanism of neuroplasticity (37), can mitigate CRCI as has been shown in prior reviews (36).

Building on this neuroplasticity-based framework, a broader range of interventions have emerged that similarly target neural adaptability. The advantageous effects of neurostimulation, neuromodulation, and sensory therapies have been tested to reduce symptoms in several conditions, such as depression, chronic pain, epilepsy, and aid post-stroke recovery (38–43).

Clinical trials have demonstrated acupuncture, and electroacupuncture (EA) has positive impact on the peripheral nervous system in cancer survivors, reducing chemotherapy-induced peripheral neuropathy (CIPN) and mitigating chemotherapy-related fatigue (44–50).

Others focused on the central nervous system by testing novel non-invasive brain stimulation (NIBS) techniques, including transcranial direct current stimulation (tDCS), transcranial alternate current stimulation (tACS), intermittent theta burst stimulation (iTBS), transcranial magnetic stimulation (TMS), and transcranial photobiomodulation (tPBM). The positive effects of NIBS on cognition have been demonstrated in healthy adults (51, 52) and in several age-related cognitive disorders (53–57), neurodegenerative diseases (58), traumatic brain injury (59–61), and other neurological disorders (62–64). Accumulating data suggest that cognitive improvement may be ascribed to NIBS-induced neuroplasticity (65); however, other cellular and physiological mechanisms—such as modulation of neurovascular coupling (66), recruitment of functional connections (67), mitochondrial ATP synthesis (68)—relevant to these interventions may also play a significant role (69). However, evidence supporting the employment of central or peripheral neurostimulation/modulation techniques in cancer survivors to rescue cognitive function remains scarce. Targeting these mechanisms through neurostimulation and neuromodulation holds promise in advancing the CRCI treatment. While several systematic reviews that focus on non-pharmacological interventions to mitigate CRCI have been published, there is no comprehensive evaluation of the existing literature on the potential cognitive benefits of non-invasive brain stimulation and other neurostimulatory and neuromodulation techniques in cancer survivors after chemotherapy.

We conducted a systematic review summarizing observations from clinical trials testing non-pharmacological interventions to mitigate CRCI via different neurostimulation and neuromodulation techniques. We sought to synthesize the impact of these techniques on cognitive outcome measures in cancer survivors treated with chemotherapy and describe the evidence corroborating their cognitive benefits based on current therapeutic protocols.

## 2 Methods

### 2.1 Data sources and search strategy

For this systematic review, we did not submit registration; however, we followed the PRISMA guidelines and searched for peer-reviewed articles in the following databases: PubMed, Embase, MEDLINE, Scopus, and PsycINFO. The following search query was created for a comprehensive database screening: ((((“transcranial electric stimulation”[Title/Abstract]) OR (“transcranial magnetic stimulation”[Title/Abstract]) OR (“cranial stimulation”[Title/Abstract]) OR (“vagal nerve stimulation”[Title/Abstract]) OR (“theta burst stimulation”[Title/Abstract]) OR (“brain stimulation”[Title/Abstract]) OR (“electroconvulsive therapy”[Title/Abstract]) OR (“transcranial direct current stimulation”[Title/Abstract]) OR (“transcranial alternating current stimulation”[Title/Abstract]) OR (“transcranial photobiomodulation”[Title/Abstract]) OR (“electroacupuncture”[Title/Abstract]) OR (“acupuncture”[Title/Abstract]))) AND (“cancer”[Title/Abstract])) AND (((“chemotherapy related cognitive impairment”[Title/Abstract]) OR (“chemotherapy related cognitive decline”[Title/Abstract]) OR (“chemotherapy induced cognitive impairment”[Title/Abstract]) OR (“chemotherapy induced cognitive decline”[Title/Abstract]) OR (“chemotherapy associated cognitive impairment”[Title/Abstract]) OR (“chemotherapy associated cognitive decline”[Title/Abstract])))) AND ((“1946/01/01”[Date - Publication]: “2025/12/31”[Date - Publication])). Our search query contained permissive terms for the intervention, such as “brain stimulation”, which allowed us to gather studies regarding deep brain stimulation or other novel methods (e.g., transcranial ultrasound brain stimulation). Since this search strategy was designed to identify original papers reporting cognitive outcome measures on patients with CRCI and any type of cancer diagnosis, we thus considered a number of methods, such as acupuncture, peripheral nerve stimulation techniques, and electroconvulsive therapy, that have neurostimulatory and neuromodulatory effects. A detailed strategy is available in the supplementary document-Search Strategy.

The search was conducted in 2025 March and April, and the publication years ranged from 1946 to December 2025, with the final search done on March 25, 2026.

### 2.2 Eligibility

The systematic search followed the PICOS framework (population, intervention, comparison, outcomes, and study design), and each point is presented in **Table 1**.

**Table 1:** Review eligibility criteria.

Elements	Description
Population (*P*)	Adult (aged ≥18 years) patients with cancer, who are receiving or have received chemotherapy without further restriction (e.g., no restriction on race, education level, income, marital status, type of cancer, progression level of disease or on chemotherapy regimen)
Intervention (*I*)	Intervention with direct effect on neurons (e.g., acupuncture, electroacupuncture, direct current stimulation, magnetic stimulation, photobiomodulation)
Control (*C*)	No restrictions
Outcome (*O*)	At least one outcome had to pertain to a cognitive domain (e.g., memory, executive function, attention) assessed as either a primary or secondary outcome. Both subjective and objective assessments are eligible, with no restrictions on the method of evaluation
Study design (*S*)	No restriction on study design

#### 2.2.1 Inclusion criteria

1. Studies were eligible if they included patients with active cancer or a history of cancer diagnosis. Inclusion and exclusion criteria were defined at the individual-study level. We did not apply restrictions on common cancer-related comorbidities (e.g., chronic kidney disease, hypertension, or cardiovascular disease).
2. Studies that included patients receiving or who received chemotherapy.
3. Studies employing interventions such as acupuncture and related techniques or other non-invasive non-pharmacological neurostimulatory or neuromodulatory intervention that has a direct impact on the central or peripheral nervous system, such as tDCS, TMS, Flexyx neurotherapy system (FNS), or sensory stimulation.
4. Studies reporting cognitive function outcomes in any domain. As cognitive decline manifests in several ways (e.g., memory loss, attention deficits, learning difficulties) with heterogeneous symptomatology, we included both objectively and subjectively measured cognitive outcomes.

#### 2.2.2 Exclusion criteria

1. Studies not reporting original results (e.g., protocol or study design papers).
2. Studies with pediatric or adolescent participants included in the study sample (<18 years old).
3. Unavailable full text (e.g., conference proceedings, abstracts).
4. Studies focused solely on physical activity, dietary, or behavioral approaches rather than the intervention of interest.
5. Non-English language publication.

We also included other systematic reviews and meta-analyses within our full-text screening phase to examine their reference lists. This allowed us to minimize the risk of omission.

### 2.3 Data extraction

Data extraction and quality appraisal were independently conducted by reviewers IL, ND, and PM. Harmonized data were incorporated into the main text; disagreements were resolved by PM. The key information extracted from each article included authors, year of publication, patient demographics, type of cancer and chemotherapy regimen, intervention protocol, cognitive domain(s) assessed, and the intervention’s impact on cognition. The comprehensive data extraction table is available in the **Supplementary Material – Table 2**.

### 2.4 Risk-of-bias assessment

The risk-of-bias assessment was performed according to the Joanna Briggs Institute (JBI) critical appraisal tools specific to case reports, case series, quasi-experimental studies, and randomized controlled trials (70). Two reviewers independently assessed bias (IL and ND). Consensus management was directed/coordinated by PM to solve disagreements regarding bias assessment. A detailed assessment is provided within the **Supplementary Material – Table 1**.

## 3 Results

The search identified 2,708 potential records, with an additional two records identified through citation searching. After removing duplicates, 1,500 articles were screened. A total of 1,422 records were excluded during the title and abstract screening as they did not meet the predefined eligibility criteria. Among these, 104 topic-relevant reviews and meta-analyses were identified, and their reference lists were screened, yielding two additional eligible studies.

A total of 78 records were sought for retrieval, of which one was not retrieved, leaving 77 records for full-text assessment. The records were screened independently by IL and ND, with disagreements resolved by PM.

**Figure 1:**
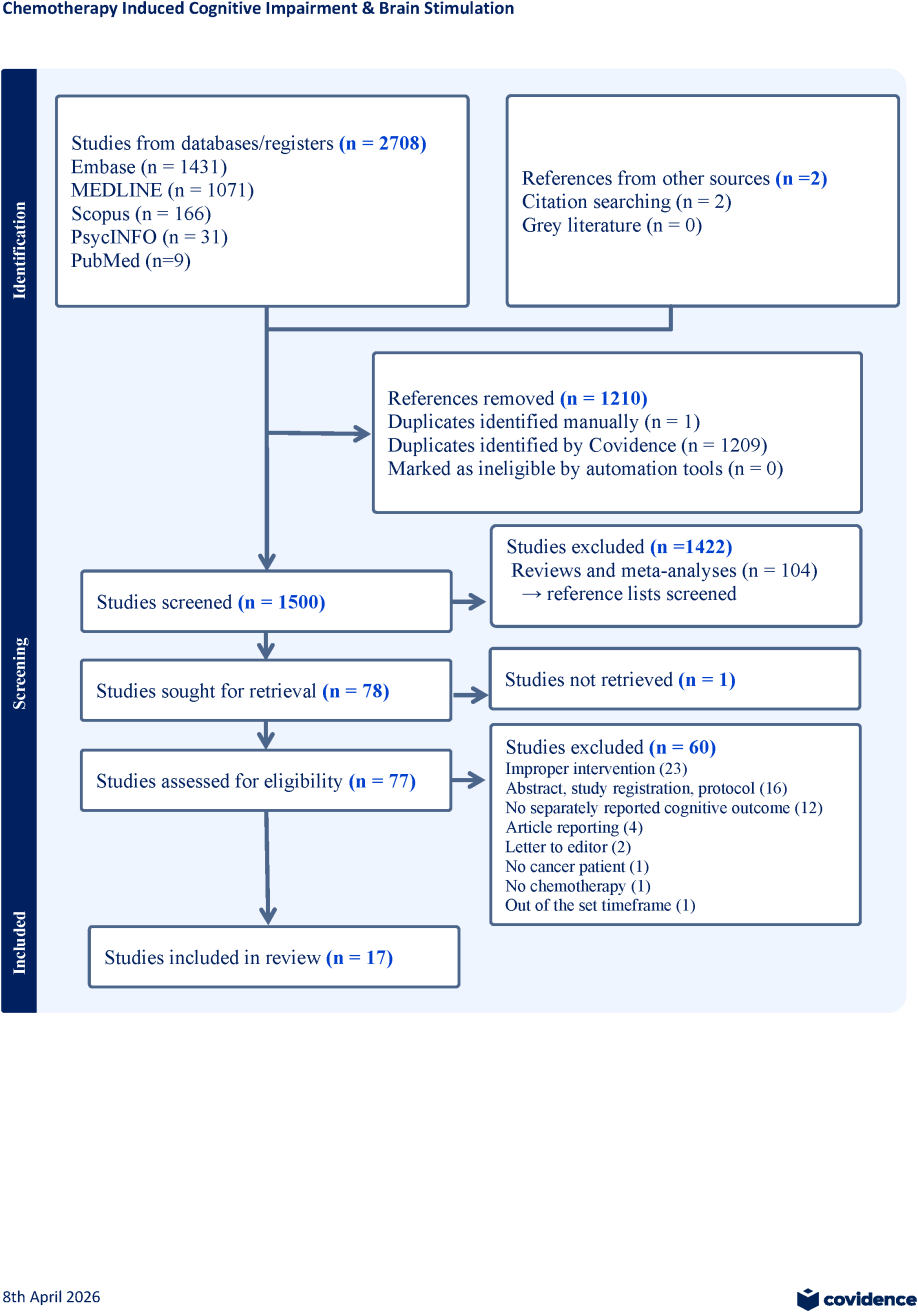
PRISMA flow diagram of study identification and screening.

The full-text screening resulted in 17 studies being included in the review: nine randomized controlled trials (52.94%), four case reports (23.53%), one case series (5.88%), two quasi-experimental studies (11.76%), and one cohort study (5.88%). The detailed screening process is presented in the PRISMA flowchart (**Figure 1**), with full data extraction provided in the **Supplementary Material – Table 2**.

The included studies present substantial heterogeneity considering the enrolled participants’ population, applied intervention types, and assessment tools. We found that self-reported cognitive function, measured by the European Organisation for Research and Treatment of Cancer Quality of Life Questionnaire Core 30 (EORTC QOL C30), was the most common outcome. Cognitive functioning is assessed through a dedicated subscale focusing on memory and concentration, providing insight into patients’ perceived cognitive difficulties (71). The questionnaire incorporates several scales, which range in score from 0 to 100. A high-scale score represents a higher response level. Thus, a high score for a functional scale represents a high/healthy level of functioning. Additionally, it incorporates four more subscales—physical, role, emotional, and social functioning—and includes several symptom scales addressing common cancer-related issues such as fatigue, pain, nausea, insomnia, and appetite loss. A high score for a symptom scale represents a high level of symptomatology/problems. However, the utilization of this tool is also presented with high heterogeneity, with several different outcome measure plans applied through different studies. Primary, secondary, and even multiple usage was reported, while it was combined with various other batteries. Specific cognitive function measures are discussed at the level of individual studies.

### 3.1 Case reports

The case reports described three different central nervous system stimulation techniques and one minimally invasive peripheral intervention (**Table 2**): TMS, tDCS, FNS, and EA. In the three non-invasive studies, the cohort included women with breast cancer. In the EA study, two cases of male participants were presented with rectal carcinoma. tDCS intervention, as reported by Knotkova et al. (72), decreased subjectively measured brain fog from 6 to 0.7 on a 0-to10 Visual Analog Scale and improved global cognition, reaching the normal range from a pre-intervention below-average level. The FNS intervention by Nelson and Esty (73) led to marked short- and long-term improvements on subjectively measured cognitive clouding. Kuo et al. (74) employed TMS intervention, which improved working memory, cognitive flexibility, and response inhibition in a breast cancer survivor. Treatment with EA, as reported by Li et al. (75), resulted in a 67-point improvement in perceived cognitive function in the first case, whereas the second case demonstrated a 33-point improvement. Two studies primarily aimed to improve cognition, accordingly Knotkova et al. (72) and Kuo et al. (74) applied objective neuropsychological batteries to assess cognition. While Nelson and Esty (73) and Li et al. (75) used subjective self-reporting instruments, cognitive function in these studies was derived from cognitive subscales of the applied tools rather than a dedicated assessment battery. Detailed descriptions of the four studies are presented in **Table 2**.

**Table 2:** Summary of case study findings.

Citation	Demographics, cancer, and chemotherapy	Intervention type	Intervention protocol	Assessed cognitive domain	Impact on cognition
Central nervous system stimulation and modulation techniques					
Knotkova, Malamud (72)	55 years old, female, breast cancer, trastuzumab, docetaxel, carboplatin	tDCS	tDCS intensity: 2 mA at 20 min/session, 5 sessions on consecutive days (1 × 1).^a^ Baseline assessment was followed by 5 sessions of tDCS on 5 consecutive days, with a 2-week follow-up	Cognitive function domains: executive functioning, memory (non-verbal), attention, global cognitive score	Global cognitive score: pre-int.: 88.7, post-int.: 108.6, follow-up: 103.9. Total memory score: pre-int.: 79.4, post-int.: 114.9, follow-up: 111.9. Executive function: pre-int: 89.5, post-int: 108, follow-up: 101.3. Brain fog: pre-int.: 6; pos-int.: 0.7
Nelson and Esty (73)	45 years old, female, breast cancer, not reported	FNS	EM stimulation of the peak EEG frequency + 20 Hz for a maximum one-second burst of alternating on/off pulsation. The patient received 10 weekly sessions of stimulation and the 11th one month later. Follow-up assessment was 6 months after completion	Brain fog—referred to as “cognitive clouding” within the article	RPFS—cognitive/mood subscale: pre-int: 6.17, post-int: 3.67; 6-month follow-up: 2.83. Current symptom ratings, cognitive clouding: pre-int: 5, post-int: 3^b^
Kuo, Chen (74)	58 years old, female, breast cancer, adjuvant chemotherapy: docetaxel, CP, tamoxifen, anastrozole, letrozole (4 cycles)	TMS	The patient received 600 iTBS pulses within a 192-s session, with repeated triplet bursts at 50 Hz for 2 s “on” (with 30 pulses) windows, followed by 8 s “off” windows, with 70% RMT. Intervention was done on 10 consecutive workdays without follow-up	Working memory, verbal memory, semantic memory, cognitive flexibility, response inhibition, subjective reporting	Working memory: fDGS: pre-int: 7, post-int: 8. bDGS: pre-int.: 8, post-int.: 8. Verbal memory—RAVLT: pre-int.: 49, post-int: 66. Semantic memory verbal fluency task: pre-int.: 33, post-int: 32. Cognitive flexibility/response inhibition—Stroop color—word interference task: pre-int.: 33, post-int.: 36
Minimally invasive peripheral nerve stimulation and neuromodulation					
Li, Yang (75)	Case 1: 66 years old, male, rectal cancer, chemotherapy: 8 cycles of CAPOX^c^ regimen; case 2: 65 years old, male, rectal cancer, chemotherapy: 8 cycles of capecitabine^d^	EA	Electroacupuncture was applied to the Baliao points: bilateral BL31, BL32, BL33, and BL34 and in combination with GV1, using a 50-Hz continuous wave for 30 min per session, twice weekly for 6 months	Perceived subjective cognitive function—EORTC QLQ-C30 cognitive function subscale	Cognitive functioning score: case 1—pre-int: 33, post-int: 100; case 2: pre-int: 17, post-int: 50

Acupuncture points: bilateral BL31 (Shangliao), BL32 (Ciliao), BL33 (Zhongliao), BL34 (Xialiao), GV1 (Changqiang). Intervention: transcranial direct current stimulation (tDCS), Flexyx Neurotherapy System (FNS), transcranial magnetic stimulation (TMS), electroacupuncture (EA). Assessment tools: Revised Piper Fatigue Scale (RPFS), forward digit span (fDGS), backward digit span (bDGS), Rey Auditory Verbal Learning Test (RAVLT), European Organisation for Research and Treatment of Cancer Quality of Life Questionnaire Core 30 (QLQ-C30) (EORTC QLQ-C30).

mA, milliampere; min, minute(s); s, second(s); Int/int., intervention; EM, electromagnetic; EEG, electroencephalograph; Hz, hertz; CP, cyclophosphamide; iTBS, intermittent theta burst stimulation; RMT, motor threshold.

^a^ The anode was placed over the left dorsolateral prefrontal cortex, point F3 of the international EEG 10–20 classification, and the cathode was over the contralateral supraorbital region. Stimulation was delivered using a transcranial direct current low-intensity stimulator (model 1300A, Soterix Medical) with two saline-soaked sponge electrodes (4.5 × 6 cm).

^b^ Current symptom ratings were not reported regarding the 6-month follow-up.

^c^ CAPOX regimen: 2 cycles of oxaliplatin at 200 mg IV on day 1; capecitabine at 1.5 g orally, twice daily on days 1–14; followed by a 7-day break; and 6 cycles with lowered oxaliplatin dosage (180 mg).

^d^ Capecitabine chemotherapy at 1.25 g orally, twice daily on days 1–14 of each 21-day cycle, followed by a 7-day drug-free interval.

### 3.2 Case series

A feasibility study conducted by Lyu, Lee (76) investigated the use of peripheral nerve stimulation to mitigate CRCI. They conducted an 8-week-long EA-based intervention involving 12 cancer survivors ≥6 months past chemotherapy. They employed objective measurement batteries and subjective questionnaires to explore cognitive performance, their primary outcome was subjective cognitive function, assessed by the FACT-Cog (V3). Objective cognitive performance was evaluated using the MoCA-K and SNSB, while an additional subjective measure, the EORTC QLQ-C30 cognitive subscale, was used to assess cognition within the broader context of quality of life. The MoCA revealed significant improvement, while FACT-Cog and SNSB demonstrated significant positive effect on specific subdomains of cognition; additional improvement was detected in recall, delayed recall and recognition. Further details are presented in **Table 3**.

**Table 3:** Summary of case series report findings.

Citation	Demographics, cancer, and chemotherapy type	Intervention type	Intervention protocol	Assessed cognitive domains	Impact on cognition
Lyu, Lee (76)	12 patients (mean age 57.3 years; 2 males, 10 females) participated. Eight had breast cancer, two thyroid cancer, one colon cancer, and one kidney cancer, with stages ranging from I to IV. All underwent surgery; nine received chemotherapy, three radiation therapy, and six hormonal or targeted therapy	EA	Acupuncture points included GV20, GV24, EX-HN1, and bilateral GB20, HT7, PC6, and KI3. Electroacupuncture was applied to HT7 and PC6 bilaterally (2 Hz, 80% intensity, 30 min) using an ES-160 stimulator. Needle depth ranged from 5–20 mm. Participants received 16 sessions (twice weekly for 8 weeks), followed by a 4-week follow-up.	Primary outcome was subjective measurement of cognition using FACT-Cog(V3). (PCI), (PCA), (IQL), (CFO) questionnaire Secondary outcomes: MoCA-K SNSB - attention, language and related functions, visuospatial functions, memory, frontal/executive functions, and other indexes EORTC QLQ- C30 physical function, role, emotion, cognition, and social function	Boston naming test: w0 = 51.7, w8 = 53.90 (*p*=>0.0207), w12 = 54.90 (*p*=0.0003) FACT-Cog: w0 = 81.17, w4 = 93.70 (*p*=0.15), w8 = 90.04 (*p*=0.33) w12 = 101.14 (*p=0.06*). PCI: w0 = 45.67, w12 = 59.33 (*p*=0.0227) PCA: w0 = 12.75, w12 = 14.56 (*p*=0.53) Total MoCA-K score: w0 = 25.50, w4 = 27.73 (*p*=0.0074) w8 = 28.40(*p*=0.0010) w12 = 28.90 (*p*=0.0003) Visuospatial/executive sections: w0 = 4.25, w4 = 4.18 (*p*=0.59), w8 = 4.6 (*p*=0.59), w12 = 4.90 (*p*=0.0368) Abstraction section: w0 = 1.5, w4 = 1.73 (*p*=0.08), w8 = 1.9 (*p*=0.0368), w12 = 1.7 (p=0.17) Delayed recall: w0 = 2.42, w4 = 4.27 (*p*=0.0007), w8 = 4.30 (*p*=0.0002), w12 = 4.40 (*p*=0.0004)

Acupuncture points: GV20 (Baihui); GV24 (Shenting), EX-HN1 (Sishencong), GB20 (Fengchi), HT7 (Shenmen), PC6 (Neiguan), KI3 (Taixi). Intervention: electroacupuncture (EA). Assessment tools: Functional Assessment of Cancer Therapy—Cognitive Function, 3rd version (FACT-Cog V3); perceived cognitive impairments (PCI), perceived cognitive abilities (PCA), impact on quality of life (IQL), comments from others (CFO), Korean version of the Montreal Cognitive Assessment (MoCA-K), Seoul Neuropsychological Screening Battery (SNSB), European Organization for Research and Treatment of Cancer Quality of Life Core Questionnaire C30 (EORTC QLQ-C30).

Int, intervention; w, week; HZ, hertz; %, percentage; min, minute(s).

### 3.3 Quasi-experimental study

We identified two quasi-experimental studies, Sawada, Zago (77) classified their work as quasi-experimental due to lack of equivalent intervention and control groups and outcomes were assessed before and after intervention. Within the context of the current review the study by Zeng, Cheng (78) can be classified as a quasi-experimental design, when focusing on CRCI participants due to small sample size and absence of randomization among participants receiving acupuncture as a subgroup of patients with gynecological cancer having CRCI (*n* = 3) identified by a separate analysis within the study. Both studies used traditional acupuncture to target the peripheral nervous system. The pilot study by Zeng, Cheng (78) tested whether cognitive functioning could improve after acupuncture treatment compared to age-, sex-, and disease stage-matched controls (*n* = 3) with gynecological cancer but preserved cognition. Importantly, controls continued with standard of care, without receiving either acupuncture or sham intervention. Zeng, Cheng (78) addressed CRCI as the primary condition and employed several objective assessment tools to measure cognition without specifically identifying any of them as primary outcome. Post-intervention cognitive function performance data were presented for groups with and without CRCI, suggesting improved performance in patients receiving active treatment, which is however not significant in this sample. Key details are presented in **Table 4**, for the purposes of this review, data relevant to the second hypothesis have been extracted and reported accordingly. Sawada, Zago (77) tested whether combined usage of relaxation with visualization and acupuncture could improve quality of life measured by EORTC QLQ-C30 as primary outcome within a mixed cancer survivor group while cognitive function was reported as secondary outcome. They demonstrated that the combined treatment improves quality of life, however they reported no significant improvement in the specific domain of cognitive function.

**Table 4:** Summary of the quasi-experimental studies and the cohort study.

Citation	Demographics (*n*, %)	Cancer type and treatment (*n*, %)	Intervention protocol	Assessed cognitive domain^a^	Impact on cognition
Zeng, Cheng (78)	Cancer patients: 15 female of 49.33 ± 9.14; age, 28–60 years old; 3 had cognitive impairment Healthy control: 15 female (age-matched), 49.60 ± 8.27 (29–59) years old	Cancer type: cervical cancer, 8 (53.3%); ovarian cancer, 1 (6.7%); uterine cancer, 6 (40.0%) Stages I–IIa: 9 (60.0%); stages IIb–IIIa: 3 (20.0%); stage IIIb: 3 (20.0%) Treatment type: surgery + chemotherapy^b^: 13 (86.7); surgery + chemotherapy^b^ + radiation: 2 (13.3)	Intervention group (3 cancer patients with CRCI) received manual acupuncture. Forehead acupuncture points: EX-HN1, EX-HN3, EX-HN5, GB8, GB15, GB20, GV20, ST8. Unilateral or bilateral insertion was determined by the acupuncturist^c^. The duration of needling was 30 min and the participants received a total of 10 sessions in 5 weeks (5 × 2). Matched controls (based on sex, age and disease stage) received usual routine care	Learning and memory: AVLT-R; information processing speed: TMT-A^d^; executive function—TMT-B^d^; attention and working memory—WAIS III Digit Span Test; verbal fluency and language comprehension—COWA, Chinese version AVLT-R	Mean scores of objective cognitive tests at post-intervention. Variables (means and SD): attention and working memory: digit span forward—Act.: 6.76 (1.94) Cont.: 6.57 (2.87); digit span backward—Act.: 2.11 (1.41), Cont.: 1.85 (1.57); verbal memory: AVLT immediate recall—Act.: 16.65 (6.45), Cont.: 16.28 (3.65); AVLT delayed recall—Act.: 5.86 (1.73), Cont.: 5.42 (3.64); AVLT recognition—Act.: 10.53 (2.98), Cont.: 10.78 (1.96); psychomotor speed—Act.: TMT-A 57.13 (27.48), Cont.: 53.80 (21.86); executive function—Act.: TMT-B 75.33 (36.07), Cont.: 74.17 (29.55); language—Act.: COWA 27.42 (6.89), Cont.: 26.76 (9.48)
Sawada, Zago (77)	Act.: 38, 24 female; age^e^ I: 3, II: 21, III: 14. Cont.: 37, 24 female, age^e^ I: 6, II: 14, III: 17	Act.: 38; GI cancer: 17 (44.7%); GYN cancer: 12 (31.6%); prostate cancer: 1 (2.6%); chemotherapy sessions^a^: 6 sessions, 35 (92.1%); ≥6 sessions, 3 (7.9%) Cont.: 37; GI cancer: 16 (43.3%); GYN cancer: 15 (40.5%); prostate cancer, 3 (8.1%); chemotherapy sessions^b^: 6 sessions, 22 (59.5%); ≥6 sessions, 11 (29.7%)	Act.: Relaxation with visualization^f^ was conducted prior to chemotherapy infusion. Acupuncture was administered for 20 min, 1/week for 6 months. Targeted acupoints: bilateral PC6, E36, BP6, and LR2; unilateral midline points RM12, RM17, and EX-HN3 BP6 The control group received standard care	EORTC QLQ-C30	No significant cognitive improvement in intervention or control group, although other QoL domains improved in the intervention group. Cognitive function—Act.: pre-in.: 72.18 ± 30.18, post-in.:74.36 ± 27.15; *p* = 0.63 Cognitive function—Cont.: pre-in.: 80.40 ± 35.58, post-in.: 76.35 ± 27.55; *p* = 0.33
Cohort study					
Chien, Yang (79)	Only active group (17); 10 female, 7 male; mean age: 65.6 ± 10.1 years	Colorectal cancer, stage III: 15, 88.2%; colorectal cancer, stage IV: 2, 11.8% FOLFOX-4: 10, 58.8%; FOLFOX-6: 7, 41.2%	Pulsed ultrasound^g^ (1 MHz, 50% duty cycle) was applied daily for 12 days, with gradual increase to elicit a *deqi* sensation^h^ for >5 min per point. Bilateral application was performed on upper extremities (PC6 and PC7) and lower extremities (BL60 and KI1) alongside oxaliplatin-based chemotherapy	EORTC QLQ-C30 cognitive functioning	Initial: 83.33 ± 27.22; pre-int. (12 days after initial): 85.00 ± 24.15; post-int.: 91.67 ± 16.20. Follow-up (30 days after intervention completion): 96.67 ± 7.03. *p* = 0.447, not significant

Acupuncture points: EX-HN1 (left and right, anterior and posterior Sishencong), EX-HN3 (Yintang), EX-HN5 (bilateral Taiyang), GB8 (bilateral Shuaigu), GB15 (Toulinqi), GB20 (Fengchi), GV20 (Baihui), ST8 (bilateral Touwei), PC6 (Neiguan), E36 (Zusanli), BP6/SP6 (Sanyinjiao), LR2 (Xingjian), RM12 (Zhongwan), RM17 (Shanzhong/Tanzhong), PC7 (Daling), BL60 (Kunlun), KI1 (Yongquan). The acupoints list is consistent with original reporting and contains duplication (E36/ST36(Zusanli), LR2/LV2; BP6/SP6). Intervention: Acupuncture, acupuncture and ultrasound combination. Assessment tools: Chinese version of the Auditory Verbal Learning Test–Revised (AVLT-R), Trail Making Test Part A (TMT-A), Trail Making Test Part B (TMT-B), Wechsler Adult Intelligence Scale–III (WAIS-III), Controlled Oral Word Association Test (COWA), European Organisation for Research and Treatment of Cancer Quality of Life Questionnaire (EORTC QLQ-C30), quality of life (QoL).

FOLFOX, fluorouracil, leucovorin, and oxaliplatin; FOLFOX-4, oxaliplatin dose of 85 mg/m^2^; FOLFOX-6, oxaliplatin dose of 100 mg/m^2^; Act., intervention/active group; Cont., control group; SD, standard deviation; MHZ, megahertz; GI cancer, gastrointestinal cancer; GYN, gynecologic and breast; pre-in., pre-intervention; post-in., post-intervention.

^a^ Cognitive domains were assessed at baseline in both group (patients with cancer and healthy controls) and post-intervention.

^b^ No further chemotherapy-related information is presented.

^c^ Acupuncture was administered by a single licensed acupuncturist trained in traditional Chinese medicine using sterile, disposable stainless steel needles (0.25 mm in diameter, 40 mm in length; Huanqiu, China).

^d^ Information processing speed was assessed using TMT-A, and executive function was assessed using TMT-B.

^e^ Age groups—I: 20–40 years old, II: 40–60 years old, III: 60–80 years old.

^f^ Relaxation with visualization was conducted prior to chemotherapy infusion, led by trained nurse researchers, and included progressive relaxation, visualization of disease and immune response, healing and recovery imagery, nature-based mental imagery, and positive emotional reinforcement.

^g^ Ultrasound device (US-750, ITO Co., Tokyo, Japan): 1.7-cm-diameter treatment head and an effective radiating area of 0.75 cm^2^.

^h^ *Deqi* sensation refers to aching, soreness, pressure, or tingling experienced when the acupuncture needle (0.25 × 40 mm) reaches an effective depth.

Further details are available in **Table 4**.

### 3.4 Cohort study

A cohort study by Chien, Yang (79) was also identified. The authors assessed the effect of a peripheral nerve stimulation protocol, combining ultrasound and acupuncture to mitigate CIPN, however no control group was included. Because their focus was on peripheral neuropathy, dedicated cognitive assessments were not included. While the EORTC QLQ-C30 includes a cognitive functioning subscale, cognitive outcome was not a primary endpoint and were only assessed as part of overall quality-of-life measures. They reported numerical improvement of cognitive function, without statistical significance at post intervention and follow-up compared to baseline. Further details are available in **Table 4**.

### 3.5 Randomized controlled trials

A total of nine randomized controlled trials (RCTs) were identified. Two of them targeted the central nervous system. Gaynor, Pergolizzi (80) evaluated transcranial direct current stimulation (tDCS) in a pilot crossover trial, while Li, Hao (81) implemented a multisensory stimulation protocol. In both studies cognitive function was a primary outcome, in accordance it was tested by several validated, domain-specific neuropsychological batteries while patient-reported questionnaires were employed to capture perceived cognitive impairment as a secondary outcome. In the tDCS study, the primary aim was to assess the feasibility, tolerability, acceptability, and efficacy of the intervention in a short 4-day long program, where half of the sessions were active stimulation in a consecutive manner, utilizing a cross over design. Active tDCS was associated with improvements in attention variability, response speed, and response accuracy compared to sham. In contrast, Li, Hao (81), showed that audiovisual training significantly improve memory, however the complex multisensory stimulation demonstrated even greater gains compared to controls.

The remaining seven studies focused on the peripheral nervous system with employing different acupuncture and EA protocols. Three studies examined the sole effect of traditional acupuncture (82–84), and three tested EA (85–87), and one investigated a combined acupuncture and EA approach (88).

Only two of these studies, Tong, Pei (82), Zhang, Man (88) specifically targeted CRCI, with cognition defined as a primary outcome and assessed using both objective neuropsychological tests and subjective measures. The remaining studies primarily focused on chemotherapy-induced peripheral neuropathy or other symptoms, with cognitive outcomes collected as secondary endpoints typically via the cognitive functioning subscale of the EORTC QLQ-C30. For example, Shen, Deng (85), Rostock, Jaroslawski (86), Chan, Lui (87) evaluated EA for CIPN and reported cognitive outcomes only as part of quality-of-life measures. Similarly, Miao, Wu (84) focused on feasibility, and Du, Tian (83) on fatigue management, with cognition assessed indirectly through patient-reported outcomes.

Detailed introduction of the samples is presented in **Table 5**, while the implemented interventions are presented in **Table 6**. Acupuncture was compared with untreated control participants by Tong, Pei (82). Following intervention, the acupuncture group scored significantly higher in cognitive tests. Zhang, Man (88) used a combination of trigeminal nerve stimulation by electroacupuncture and body acupuncture (EA/TNS+ BA) to mitigate CRCI and compared this to minimum acupuncture stimulation (MAS). Their primary cognitive outcomes were obtained by the MoCA test, which was not significantly different between the groups pre- to post-intervention, however marked improvement was reported in attention and working memory function scores on Reverse Digit Span Test within the combination group.

**Table 5:** Characteristics of the samples of the included randomized trials.

Citation	Demographics (*n*, %)	Cancer type and chemotherapy
Central nervous system and sensory stimulation		
Gaynor, Pergolizzi (80)	16 female patients between 40 and 65 years old	Breast cancer; chemotherapy was completed at least 6 months prior to intervention; without restriction regarding the current reception of endocrine therapy
Li, Hao (81)	80 female patients between 18 and 60 years old	Breast cancer; two chemotherapy cycles with at least 6 ACT regimen
Peripheral nervous system stimulation		
Tong, Pei (82)	75 patients; treatment group (39) mean age: 43.11 ± 4.23; control group mean age: 42.26 ± 4.42; gender not reported	Breast cancer; total duration of chemotherapy: 3–6 months; chemotherapy regimen, *n* (%); treatment group: TC regimen: 19 (48.7%), TP regimen: 9 (23.1%), ECx4-Tx4: 11 (28.2%); control group: TC regimen: 18 (50%), TP regimen: 7 (19.4%), ECx4-Tx4: 11 (30.6%)
Zhang, Man (88)	92 patients; average age: EA/TNS+BA: 47.9 ± 9.7 years; MAS: 47.9 ± 10.6 years; all female	Breast cancer; FEC-T regimen: EA/TNS+BA, *n* = 9 (20.0%); MAS, *n* = 11 (23.4%). TAC regimen: EA/TNS+BA, *n* = 7 (15.6%); MAS, *n* = 7 (14.9%). TC regimen: EA/TNS+BA, *n* = 12 (26.7%); MAS, *n* = 9 (19.0%). AC regimen: EA/TNS+BA, *n* = 2 (4.4%); MAS, *n* = 0 (0%). TPH regimen: EA/TNS+BA, *n* = 11 (24.4%); MAS, *n* = 15 (31.9%). Others: EA/TNS+BA, *n* = 4 (8.9%); MAS, *n* = 5 (10.6%)
Rostock, Jaroslawski (86)	60 patients; EA: mean age, 49.9 (SD: 9.6) years; 10 female, 4 male. HB: mean age, 52.3 (SD: 11.3) years; 12 female, 1 male. Vit B: mean age, 56.3 (SD: 11.1) years; 10 female, 5 male. Placebo: mean age, 52.0 (SD: 8.1) years; 14 female, 3 male	EA: Breast cancer, *n* = 6 (42.9%); ovarian cancer, *n* = 3 (21.4%); lymphoma, *n* = 4 (28.6%); other: *n* = 1 (7.1%); secondary cancer, *n* = 2 (14.3%). HB: Breast cancer, *n* = 3 (23.1%); ovarian cancer, *n* = 3 (23.1%); lymphoma, *n* = 5 (38.5%); other, *n* = 2 (15.4%); secondary cancer, *n* = 3 (23.1%). Vit B: Breast cancer, *n* = 4 (26.7%); ovarian cancer *n* = 4 (26.7%); lymphoma, *n* = 6 (40.0%); other, *n* = 1 (6.7%); secondary cancer, *n* = 4 (26.7%). Placebo: Breast cancer, *n* = 8 (47.1%); ovarian cancer, *n* = 3 (17.6%); lymphoma, *n* = 2 (11.8%); other, *n* = 4 (7.1%); secondary cancer, *n* = 2 (23.5%). Chemotherapy—EA: vinca alkaloids, *n* = 4 (28.6%); platin derivatives alone, *n* = 1 (7.1%); taxanes alone, *n* = 6 (42.9%); platin derivatives and taxanes combined, *n* = 3 (21.4%); total number of different cytostatics, 2.1 ± 1.4; number of different neurotoxic cytostatics only, 1.6 ± 1.2. HB: vinca alkaloids, *n* = 5 (28.5%); platin derivatives alone, *n* = 2 (15.4%); taxanes alone, *n* = 3 (23.1%); platin derivatives and taxanes combined, *n* = 3 (23.1%); total number of different cytostatics, 1.5 ± 0.9; number of different neurotoxic cytostatics only, 1.1 ± 0.3. Vit B: vinca alkaloids, *n* = 6 (40.0%); platin derivatives alone, *n* = 0 (0%); taxanes alone, *n* = 4 (26.7%); platin derivatives and taxanes combined, *n* = 5 (33.3%); total number of different cytostatics, 1.7 ± 1.0; number of different neurotoxic cytostatics only, 1.2 ± 0.4. Placebo: vinca alkaloids, *n* = 3 (17.6); platin derivatives alone, 3 (17.6%); taxanes alone, *n* = 8 (47.1%); platin derivatives and taxanes combined, *n* = 5 (29.4%); total number of different cytostatics, 2.0 ± 0.8; number of different neurotoxic cytostatics only, 1.3 ± 0.6
Chan, Lui (87)	Male: EA: 18 (33%), S: 15 (27%). Female: EA: 9 (16%), S: 13 (24%). Cancer stage—stage III EA: 24 (44%), S: 24 (44%); stage IV EA: 3 (6%), S: 4 (7%). Age: EA: 60.0 ± 8.57 years old, S: 62.5 ± 7.62 years old	Colorectal carcinoma; oxaliplatin-based chemotherapy. Chemotherapy regimen—XELOX EA: 24 (44%), S: 26 (47%); FOLFOX or other EA: 3 (6%), S: 2 (4%)
Shen, Deng (85)	4 groups, total (152); all female mean age, 53.30 ± 8.94 years. 2 Hz EA: (39); age: 53.74 (8.44). 100 Hz EA: (38); age: 54.39 (8.25). 2/100 Hz EA: (37); age: 52.02 (9.62). Mecbl: (38); age: 53.02 (9.61)	Breast cancer patients; Only reported that the proportions of patients with metastatic disease, chemotherapy cycles, chemotherapy protocols, with no significant differences across groups,
Miao, Wu (84)	*N* = 70; int. (35), cont. (35). Age (years): int. 52.17 ± 4.38; cont. 52.34 ± 5.14. Sex: int. 18 female, 17 male; cont. 15 female, 20 male	Advanced gastric cancer—Int. 35: stage III; Cont. 35: stage III. Chemotherapy: 2 cycles; capecitabine: 1,650 mg/m^2^/day orally on days 1–14. Paclitaxel: 175 mg/m^2^ IV on day 1
Du, Tian (83)	Total enrolled (61) Int. (26); 8 females, 18 males; mean age 55.62 ± 12.04 years Cont. (24); 9 females, 15 males; mean age 61.83 ± 10.55 years	Colorectal cancer—FOLFOX, FOLFIRI, or XELOX, administered over two cycles (total duration: 6 weeks)

Interventions: Electroacupuncture trigeminal nerve stimulation plus body acupuncture (EA/TNS+BA); minimal acupuncture stimulation (MAS); electroacupuncture (EA); hydroelectric baths (HB); vitamin B complex (Vit B); sham/placebo (S). Chemotherapy regimens: Epirubicin + cyclophosphamide + docetaxel (ACT); docetaxel + cyclophosphamide (TC); paclitaxel + carboplatin (TP); epirubicin + cyclophosphamide ×4 followed by docetaxel ×4 (EC×4–T×4); fluorouracil + epirubicin + cyclophosphamide followed by docetaxel (FEC-T); docetaxel + doxorubicin + cyclophosphamide (TAC); doxorubicin + cyclophosphamide (AC); docetaxel + carboplatin + trastuzumab (TPH); capecitabine (Xeloda) + oxaliplatin (XELOX); folinic acid + fluorouracil + oxaliplatin (FOLFOX); methylcobalamin (Mecbl); irinotecan+leucovorin+ fluorouracil (FOLFIRI).

Other: sample size and percentage (*n* =;%); standard deviation (SD); intravenous (IV); control (cont.); intervention (int.).

**Table 6:** Summary of interventions in randomized trials.

Citation	Intervention group protocol	Control group protocol	Intervention frequency, duration, and follow-up
Central nervous system and sensory stimulation			
Gaynor, Pergolizzi (80)	Electrodes were placed at F3 (anode) and F4 (cathode) per the 10–20 EEG system. The tDCS device ramped up over 30 s before the CPT task and applied 1 mA stimuli for 15 min through active sessions	Protocol was identical to active sessions, except that no 1-mA stimulation was applied for 15 min after the initial ramp-up phase	The participants completed four consecutive daily sessions (two active, two sham), each lasting 30 min, with no follow-up
Li, Hao (81)	The multisensory group received audiovisual training, scalp tactile stimulation, object-based tactile training, and olfactory stimulation. Scalp tactile stimulation included temple massage (clockwise/counterclockwise directions), pressure application from the glabella to Baihui, then down to Fengchi acupoint, and finger combing from the forehead to the back. Tactile training involved exploring objects of various sizes, temperature, and textures with both hands inside a box. Olfactory training involved memory stimulation by smelling two essential oils (20 s each), with a recall test after 5 min, and bedtime stimulation with a lavender-scented candle, relaxing music, and 10 min of diaphragmatic breathing. Audiovisual training was identical to that of the control group	Audiovisual group: Audiovisual training was based on the 66nao Brain Training system (Wispirit Tech) and included photo–name matching module to improve face recognition and delayed recall, listen and link module to train semantic processing and working memory, and the sequence memory module to improve memory and execution skills	Each treatment cycle lasted 26 days, including 5 days of hospitalization for routine care and 21 days of rest at home. Stimulation training was conducted daily for 20 min and repeated across four cycles. Tactile and olfactory training were conducted daily for 10 min over 5 days, repeated in four cycles. The researchers maintained contact with the participants and their supervising family members via a WeChat group
Peripheral nervous system stimulation			
Tong, Pei (82)	Acupuncture was used on the following acupoints: DU20, EX-HN1, and KI3; other acupoints could also be stimulated (reflecting symptomology) as follows: DU24, KI4, GB39, and ST36; a needle was inserted 25–35 mm deep to elicit a “deqi” sensation^a^	No acupuncture	Patients underwent 2 cycles of 4-week acupuncture treatment, separated by a 3-day break. Treatment was provided 5 days per week with 2 days of rest. No follow-up assessment was conducted
Zhang, Man (88)	For EA/TNS + BA: manual stimulation of: Bilateral: (HT7), (TE5), (ST36), (ST40) and (SP6) Midline: (CV12), (CV4), (GV26) Electrical stimulation: Midline: GV20 (+), EX-HN3 (−), Left: EX-HN1 (−) and GB15(+), Right: EX-HN1 (−), and GB15 (+), Bilateral: GB8 (L+, R−), EX-HN5 (L+, R−), ST8(L+, R−) Peak output of the device: 6 V and 48 mA (ITO ES-360); the frequency and phase duration for 30 min was 2 Hz and 100 μs, respectively	MAS: 6 acupoints Bilateral: (LI10), (BL59) and (BL7); electrical stimulation was only performed on BL17 (L+, R−); stimulation was performed to a level at which “patients just started feeling stimulation”	The intervention was conducted twice weekly for 8 weeks, with 30 min of electrical stimulation and 30-min needle retention at body acupoints
Rostock, Jaroslawski (86)	EA group: CIPN patients with upper and/or lower extremity symptoms received EA (50 Hz electrostimulation) on symptom-related acupoints: Legs: LV3, SP9, GB41, GB34 Arms: LI4, SI3, HT3 Needle depth was adjusted to elicit a “deqi” sensation^a^	HB: Patients immersed their arms or feet (up to a hand’s width above the elbow or ankle) in 35 °C water, which acted as an electrode. Each 15-min session applied 50 Hz of faradic current via cross-galvanization to each extremity, adjusted to the individual’s tingling tolerance Vit B: Patients—100 mg thiamine nitrate and 100 mg pyridoxine hydrochloride Placebo: Identical lactose capsules (to Vit B, in form, taste, and odor)	EA was administered in 8 ± 1 session for 15 min. HB contained 8 ± 1 sessions, which lasted for 15 min. Vit B: Th patients took 3 capsules daily for 3 weeks. Placebo was taken 3 times daily
Chan, Lui (87)	Electrical stimulation was applied using continuous 2-Hz waves (200-ms pulse width) at a variable intensity (2–5 mA), adjusted to each patient’s minimum perceptible sensation. The chosen acupuncture points were LI4, PC6, LI11, Baxie, ST36, SP6, LR3, and Bafeng^b^	The sham group received stimulation on the following acupoints: LI4, PC6, LI11, Baxie, ST36, SP6, and LR3. The needles were not inserted, only adhered to the skin by a small plastic ring, and EA shaming was reached by connecting the needles to the incorrect output socket of the device	The EA/S intervention was performed weekly (1 × 1) and for 25 min/session over 12 consecutive weeks and was followed by a reassessment after 12 weeks of completion
Shen, Deng (85)	EA was delivered at 2 Hz, 100 Hz, or alternating 2/100 Hz, with intensity 0.5–4 mA, adjusted to tolerance Standardized bilateral upper and/or lower limb acupoints; patients with whole-limb symptoms received both protocols Upper limb: LI11, SJ5, LI4, SI3, EX-UE9; Lower limb: GB34, ST36, SP9, SP6, LR3, EX-LE10; EA pairs; Upper limb: LI4–SJ5; Lower limb:ST36–SP6	Routine care, for 4 weeks—Mecbl tablets, orally, 1 tablet at a time, 3 times and followed up for 4 weeks	EA group received 12 sessions (30 min/session, 3/week) over 4 weeks. With a 4-week follow-up
Miao, Wu (84)	Acupuncture with adjunct moxibustion during conventional treatment Moxibustion: Gentle heat was provided by burning the 2-cm moxa on needle handle 2 to 3 cm above the skin Acupoints: CV4, CV6, ST36, SP15, PC6, SP10, SP8, ST28, ST29	The patients received routine care consisting of neoadjuvant chemotherapy with capecitabine and paclitaxel combined with radiotherapy, followed by regular clinical monitoring, including blood tests and assessment of liver and kidney function	Acupuncture was delivered during the chemoradiotherapy period, with sessions lasting ~20 min each. Without further specification
Du, Tian (83)	Reinforcing acupuncture; acupoints: unilateral CV6, CV4, and bilateral ST36. The reinforcing technique consisted of light rotation combined with heavy thrusting and gentle lifting, using low-frequency, small-amplitude movements from superficial to deeper tissue layers	Chemotherapy was given for two cycles, 6 weeks in total	Needles were retained for 30 min per session. Acupuncture was administered concurrently with chemotherapy over two cycles (6 weeks total). Each cycle included four sessions: one session on the day before chemotherapy and additional sessions on days 1, 2, and 3 of chemotherapy, resulting in a total of eight sessions across two cycles

Acupuncture points: DU20/GV20 (Baihui)—DU20 and GV20 refer to the same Baihui acupoint (103, 104), EX-HN1 (Sishencong), KI3 (Taixi), DU24 (Shenting), KI4 (Dazhong), GB39 (Juegu), ST36 (Zusanli), LI4 (Hegu), PC6 (Neiguan), LI11 (Quchi), SP6 (Sanyinjiao), LR3 (Taichung), LV3 (Taichong), SP9 (Yinlingquan), GB41 (Zulingqi), GB34 (Yanglingquan), SI3 (Houxi), HT3 (Shaohai), HT7 (Shenmen), TE5 (Waiguan), ST40 (Fenglong), CV12 (Zhongwan), CV4 (Guanyuan), GV26 (Shuigou), EX-HN3 (Yintang), GB15 (Toulinqi), GB8 (Shuaigu), EX-HN5 (Taiyang), ST8 (Touwei), BL17 (Tongtian), LI10 (Shousanli), BL59 (Fuyang) EX-UE9 (Baxie), EX-LE10 (Bafeng), CV6 (Qihai), SP15 (Daheng), SP10 (Xuehai), SP8 (Diji), ST28 (Shuidao), ST29 (Guilai). The acupoints list is consistent with original reporting and contains duplication (LV3/LR3 (Taichung–Taichong)). Intervention: Transcranial direct current stimulation (tDCS); Conners’ Continuous Performance Test II (CPT-II). Electroacupuncture trigeminal nerve stimulation plus body acupuncture (EA/TNS+BA); minimal acupuncture stimulation (MAS); electroacupuncture (EA); hydroelectric baths (HB); vitamin B complex (Vit B); sham (S); methylcobalamin tablets (Mecbl).

EEG, electroencephalography; s, seconds; min, minute(s); mA, milliampere(s); stim., stimulation; Hz, hertz; CIPN, chemotherapy-induced peripheral neuropathy; +, positive electrode; −, negative electrode; L, left; R, right.

^a^ *Deqi* sensation refers to aching, soreness, pressure, or tingling experienced when the acupuncture needle (0.25 × 40 mm) reaches an effective depth.

^b^ Baxie and Bafeng acupoints were optional depending on injury status. Treatment was delivered by a registered Chinese medicine practitioner with more than 5 years of clinical experience.

Among studies with cognition as a secondary outcome, findings were mixed. Rostock et al. (86), in a four arm study, comparing EA, hydroelectric baths (HB), Vitamin B (Vit B), and placebo reported non-significant trends toward improved cognitive function in the EA and hydrotherapy groups (86). Chan et al. (87) and Shen et al. (85) found no significant cognitive changes following EA interventions (85, 87). In a feasibility study, Miao, Wu (84) reported a significant between-group difference in post-intervention quality of life, favoring the acupuncture group; however, the absence of pre–post comparisons limits interpretation of treatment-related change. Lastly Du, Tian (83), tested acupuncture in relation of fatigue management, and reported significant within group improvement, in the acupuncture group, on the Piper fatigue scale, although these findings were not corroborated by EORTC QLQ-C30 results.

An extended presentation of effect on cognition is presented in **Table 7**.

**Table 7:** Summary of the cognitive results in randomized trials.

Citation	Assessed cognitive domains and assessment tools	Impact on cognition
Central nervous system and sensory stimulation		
Gaynor, Pergolizzi (80)	Attention (reaction time and accuracy) CPT-II	PAOFI scores: (*F*[1,13] = 2.28, *p* = 0.155) SGI scores: (*F*[1,13] = 3.17, *p* = 0.098) CPT active stimulation reduced Hit RT SE vs sham (*p* < 0.05) Decreased change in reaction time (ISIs, *p* < 0.01)
Li, Hao (81)	Memory, cognitive deficit, executive function RBMT-II^a^, BADS^b^	Multisensory group scored significantly higher on RMBT-II total and all subtests versus baseline and outperformed the audiovisual group on total score and 11 of 12 subtests (*p* < 0.05), except message recall. The audiovisual group improved from baseline on total score and most subtests, except picture recognition, immediate story recall, date, and immediate route recall. For BADS, the multisensory group significantly outperformed the audiovisual group on total score and all subtests except temporal judgment and zoom map (*p* < 0.05). Both groups improved from baseline on four subtests (excluding temporal judgment and zoom map) and total score (*p* < 0.01 audiovisual; *p* < 0.001 multisensory)
Peripheral nervous system stimulation		
Tong, Pei (82)	Functionality—FACT-Cog V3: PCA, PCI, QOL, OTH; short-term memory—AVLT1; delayed recall—AVLT2; recognition—AVLT3; long-term memory—VFT; attention, cognitive processing speed, and visual working memory—SDMT; executive function—CDT and TMT-B; general cognitive status—MMSE	The treatment group showed significant improvements after acupuncture on FACT-Cog, AVLT3, and CDT scores compared to baseline (paired *t*-test, *p* < 0.05), while the control group showed no significant changes at post-assessment FACT-COG: *p* = 0.001, PCI: *p* = 0.027, QoL: *p* = 0.279, OTH: *p* = 0.697, PCA: *p* = 0.014, AVLT1: *p* = 0.873, AVLT2: *p* = 0.936, AVLT3: *p* = 0.002, VFT: *p* = 0.642, SDMT; *p* = 0.269, CDT: *p* = 0.002, TMT-B: *p* = 0.901
Zhang, Man (88)	Primary outcome: MoCA Secondary outcome: attentional function and working memory: DGS Quality of life: EORTC QLQ-C30, BR23 (V3)	Significant group effects were observed only for the reverse digit span (*F* = 7.030, *p*= 0.009). Between-group comparisons showed that the EA/TNS + BA group scored significantly higher than the MAS group on the reverse digit span at week 2 (*p*= 0.045) and week 8 (*p*= 0.004); the respective effect size were 0.53 and 0.48
Rostock, Jaroslawski (86)	Quality of life; cognitive function—secondary outcome EORTC QLQ-C30 scale	Mean ± SD Day 0—EA: 66.7 ± 23.6, HB: 39.7 ± 30.1, Vit B: 62.2 ± 28.5, Placebo: 67.6 ± 24.6, Sum: 59.9 ± 28.2 Day 21—EA: 70.2 ± 18.7, HB: 55.1 ± 30.0, Vit B: 71.1 ± 24.0, Placebo: 79.4 ± 18.2, Sum: 69.8 ± 23.9 Day 84—EA: 76.7 ± 21.8, HB: 58.5 ± 36.2, Vit B: 61.6 ± 25.8, Placebo: 71.0 ± 13.2, Sum: 67.2 ± 25.2
Chan, Lui (87)	Quality of life; cognitive function—secondary outcome EORTC QLQ C-30	Cognitive function (mean ± SD) 1st cycle: EA (85.2 ± 16.23) vs S (86.3 ± 16.39) between group *p* = 0.396 2nd cycle: EA (91.4 ± 13.37) vs S (88.1 ± 16.27) between group *p* = 0.228 3rd cycle: EA (88.3 ± 12.07) vs S (89.3 ± 12.18) between-group *p* = 0.565 Week 12: EA (87.7 ± 13.55) vs S (88.1 ± 14.24) between-group *p* = 0.957 Week 24: EA (77.8 ± 24.02) vs S (83.9 ± 17.26) between-group *p* = 0.233
Shen, Deng (85)	Quality of life; cognitive function—secondary outcome EORTC QLQ C-30	No significant within-group changes were observed in any EA group at week 4 or week 8 (all *p* > 0.05). Mean changes were minimal across groups (week 4: all ~0.0; week 8: 0.0–0.0)
Miao, Wu (84)	Self-reported quality of life without further specification^c^	Int. group compared to the cont. group (78.45 ± 5.85 vs 61.34 ± 6.27), *p* < 0.001
Du, Tian (83)	Secondary outcomes: PFS cognition dimension; EORTC QLQ-C30 cognitive function	PFS showed a significant improvement in the treatment group: per-int., 4.61 ± 1.43; post-int.: 3.86 ± 1.15 (*p* < 0.05). In contrast, the control group demonstrated no significant change (3.65 ± 1.92 to 3.81 ± 1.83, *p* > 0.05). EORTC QLQ-C30 showed no statistically significant changes in either group. The treatment group scores increased from 66.67 (20.84) to 69.23 (16.79), while the control group scores decreased slightly from 66.67 (33.33) to 63.89 (26.77), with both changes not reaching statistical significance

Interventions: Electroacupuncture trigeminal nerve stimulation plus body acupuncture (EA/TNS+BA), minimal acupuncture stimulation (MAS), electroacupuncture (EA), hydroelectric baths (HB), vitamin B complex (Vit B), sham (S), summarized score (Sum). Abbreviations/assessment tools: Conners’ Continuous Performance Test II (CPT-II); Patient Assessment of Own Functioning Inventory (PAOFI); Sensory Gating Inventory (SGI); variability in response speed (Hit RT SE); interstimulus intervals (ISIs); Rivermead Behavioral Memory Test, second edition (RBMT-II); Behavioral Assessment of the Dysexecutive Syndrome (BADS). Functional Assessment of Cancer Therapy–Cognitive Function, version 3 (FACT-Cog V3); perceived cognitive abilities (PCA); impact of perceived cognitive impairments on quality of life (IQL); quality of life (QoL/QOL); comments from others (OTH); perceived cognitive impairments (PCI); Auditory Verbal Learning Test—immediate recall (AVLT1); Auditory Verbal Learning Test—delayed recall (AVLT2); Auditory Verbal Learning Test—recognition (AVLT3); verbal fluency test (VFT); Symbol Digit Modality Test (SDMT); Clock Drawing Test (CDT); Trail Making Test, part B (TMT-B); Mini-Mental State Examination (MMSE); Montreal Cognitive Assessment (MoCA); Digit Span Test (DGS; forward and backward); EORTC QLQ-C30 Breast Cancer Module, version 3 (EORTC QLQ-BR23 V3); European Organization for Research and Treatment of Cancer Quality of Life Core Questionnaire C30 (EORTC QLQ-C30); Piper Fatigue Scale (PFS) , based on a provided flowchart.

Int., intervention group; Cont., control group; pre-int., pre-intervention; post-int., post-intervention.

^a^ The RBMT-II comprises 12 subtests (remembering names, hidden belongings, appointments, picture recognition, face recognition, immediate and delayed story recall, orientation, date, immediate and delayed route recall, and message recall). Total scores range from 0 to 24, with lower scores indicating poorer memory performance.

^b^ The BADS consists of six subtests (rule-shift cards, action program, key search, temporal judgment, zoo map, and modified six elements), each scored from 0 to 4, yielding a total score range of 0–24, with lower scores indicating greater executive dysfunction.

^c^ Cognitive function was evaluated only at the end of the intervention.

### 3.6 Risk-of-bias assessment

The four case reports included in this review provide high quality reporting based on the JBI’s case report criteria (72–75).

The single-arm trial of Lyu et al. (76) evaluated the feasibility of EA treatment and was appraised following case series requirements. The screening procedure described in their paper suggests that the inclusion of participants was not consecutive and complete. The reported *p*-values are ambiguous, as the statistical analysis section lacks sufficient detail to clarify whether the analysis was tailored for data normality or within-group comparisons across multiple follow-up points and whether adjustments for multiple comparisons were applied.

In the paper of Zeng et al. (78), participant characteristics were not reported separately for cases with neoplastic disease and cognitive impairment and cognitively intact age- and disease-stage matched controls. Therefore, we could not determine the statistical significance of the difference based on the sample size of three per group. The second quasi-experimental study by Sawada et al. (77) is limited by self-selection into groups, absence of randomization and blinding, and unequal intensity of care between groups. Substantial and differential attrition, particularly in the control group, further threatens validity. Additionally, the combined intervention (acupuncture with relaxation/visualization) prevents isolation of the independent effect of acupuncture.

Inferences from the cohort study by Chien et al. (79) should be interpreted cautiously due to its single-arm design without a comparison group. Additionally, cognitive outcomes were assessed as secondary measures.

Eight RCTs followed a parallel design with separate treatment groups (81–88), while one study (80) followed a randomized crossover design where each participant served as their own control. Therefore, several criteria are not applicable for this tDCS study (80). Relevant clinical information was reported across all articles except for Gaynor et al. (80), which did not include the demographic characteristics for the final sample.

Participant allocation to randomized groups was concealed in two studies (86, 88). Concealment was not specified in six other included articles (81–85, 87). Complete blinding of treatment deliverers was not reported in any randomized trials. Partial blinding of deliverers was achieved in the study of Rostock et al. (86) for the placebo and high-dose Vit B supplementation subgroups but could not be implemented for the HB and EA intervention arms of this study, and it was not applicable in the tDCS study (80). Assessor blinding was achieved in four RCTs (82, 85, 87, 88) and was not specified for Li et al. (81) and Miao et al. (84). Since cognitive outcomes were obtained using objective assessments, unblinding outcome assessors did not increase the risk of bias in the study of Gaynor et al. (80). This criteria was also not applicable for the study of Rostock et al. (86), given that they utilized the EORTC test which is independent from the assessor. Participant blinding was achieved in three studies: it was complete and successful in case of Zhang et al. (88), but its fidelity was not checked by Chan et al. (87) and was partial in another RCT (86).

The outcomes of interest at baseline were reported and similar between groups in studies with two treatment arms, but the similarity of the treatment groups in terms of cognitive status is unclear in the case of one RCT (86). Appropriate statistical analyses were reported by eight studies (79, 82–88). Despite the fact that a sufficient sample size (*n* = 40/group) enrolled in the study of Li et al. (81), the inter- and intra-group comparisons were performed using only independent and paired-samples *t*-tests instead of ANOVA and without checking the violations of these tests. The statistical analysis plan adopted by Gaynor et al. (80) was appropriate for the randomized crossover design, assuming that data is normally distributed and the variance is homogeneous, which was not reported. Finally, follow-up was reported properly in all RCTs. The exception was Tong et al. (82) in which they report some data loss to follow-up, but they do not provide further information on this aspect.

## 4 Discussion

Advances in cancer therapy have led to higher survival rates, which, in turn, have heightened attention on managing long-term side effects (2, 3), such as chemobrain, a significant clinical problem gaining increasing attention. Current oncology guidelines emphasize integrating psychosocial support and personalized exercise programs into treatment protocols to improve quality-of-life (QoL) adherence, cancer-related anxiety, stress, and depression (89, 90). Non-pharmacological approaches have gained interest in the context of CRCI, including dietary or behavioral interventions which prior reviews have described (91, 92). However, neurostimulation and neuromodulation techniques, which have been shown to have cognitive benefits in other conditions, have limited application in a post-chemotherapeutic setting. This systematic review assessed the literature reporting the impact of central or peripheral neurostimulation and neuromodulation approaches on CRCI based on original data, a field yet in its infancy. These interventions—albeit through different mechanisms—specifically target the nervous system and have the potential to mitigate neuropathologies implicated in the genesis of CRCI, a condition with perplexing pathophysiology (7).

In the current review, acupuncture and EA demonstrated benefits across various conditions, including cancer-related fatigue, nausea, and neuropathy, and may improve cognitive function (93). Additionally, in vascular cognitive impairment mitigation, primary acupuncture use and its combination with routine pharmacological treatment yielded superior results compared to standard care (94). Our research found ambivalent support for acupuncture adaptation in CRCI management, as five studies reported (76, 82–84, 88) a significant improvement in outcomes following intervention. Although Rostock et al. (86) and Chan et al. (87) reported promising results, these are nevertheless less robust than the other four studies’ findings, underscoring the need for further research with larger sample sizes and standardized intervention and assessment protocols to confirm efficacy and ensure reproducibility (for details, see **Table 7**). The sum of the sample sizes for these studies is 359, and given that two studies used EORTC QLQ-C30 in the 12 weeks of follow-up, these cognitive outcomes could be synthesized. However, the analysis of the combined sample did not reveal a significant difference between the active and sham treatment groups. Electroacupuncture conferred significant cognitive benefits according to the study of Li et al. (75); however, these results were not was strengthened by another recent randomized study (85). Taken together, targeting the peripheral nervous system may alleviate cognitive deficits in cancer survivors, but the evidence is weak due to the subjective nature and timeline of cognitive assessment in the underlying studies and bias in study design. Improved reporting practices are also needed to more reliably assess the potential of acupuncture studies—which demonstrate therapeutic effects regarding CIPN and other chemotherapy-associated symptoms—in mitigating CRCI, which is required to be included in systematic reviews and meta-analyses (95).

There is increasing evidence that NIBS like TMS and tDCS may have positive impacts on neurophysiological processes supporting cognition (65, 96–98). In line with these results, we identified a case report by Knotkova et al. (72) in which tDCS intervention improved cognitive function, which gained further support from a pilot randomized crossover trial by Gaynor et al. (80), in which intervention led to self-reported cognitive improvement. During active sessions, reaction time variability decreased compared to that of sham sessions. A positive effect of TMS was reported in one case report by Kuo et al. (74). However, the role of these modalities in chemotherapy-related pathologies requires further investigation, including the development of mechanism-based trials. Additional studies are needed to define the durability of their beneficial effects and to determine the optimal intervention schedule in relation to chemotherapy, including timing (during vs after treatment), duration, and frequency. Sensory stimulation has also gained substantial attention in the context of promoting healthy aging, delaying cognitive decline, and preventing dementia. Among the most prominent modalities are visual and tactile stimulation, as demonstrated by (99). Beneficial effects of olfactory training regarding cognitive function in older adults, as well as patients with dementia and mild cognitive impairment, have been reported in randomized trials by (100, 101). Notably, Kim et al. (102) conducted a randomized sham-controlled preclinical study and found that gamma entrainment—a 40-Hz modulated sensory stimulation—significantly improved short-term memory, attention, and executive function in a validated mouse model of chemobrain. In alignment with these observations, Li et al. (81) reported significantly better outcomes in their multisensory group despite significant improvements that were also observed in the audiovisual training group. In total, these studies included 59 participants; however, the sample was heterogeneous and unevenly distributed across studies.

### 4.1 Limitations

The search strategy was designed to comprehensively capture the most relevant studies; however, restricting inclusion to English-language articles represents a limitation. As to the current state of art, the included studies provide limited evidence, given their pilot nature and/or research design, with none of them sufficiently powered to confirm a positive effect of the tested NIBS techniques. Sensory stimulation appeared to demonstrate potentially efficacious results, but generalized application requires further investigations. Acupuncture and EA also gained considerable attention in our included studies, as both methods were tested in RCTs; however, intervention protocols (stimulated acupuncture points, duration), assessed cognitive domains, and assessment tools presented substantial heterogeneity. In particular, the subjective and objective measures of CRCI often show weak or inconsistent agreement. Thus, the observed treatment effects should be interpreted cautiously. Based on the positive experiences, these studies merit further testing, with harmonized cognitive domain assessment, substantial participant numbers, and extended follow-up periods.

## 5 Conclusion

Apart from a few high-quality case reports, case series, and experimental studies, only nine pilot RCTs that provide initial evidence on the feasibility and effectiveness of intervention protocols within the scope of this review have been completed. The identified studies presented different methodologies, with EA as the most frequently utilized intervention in the included RCTs. However, all interventions need further randomized testing in a sufficiently large cancer population to establish good practices in this highly vulnerable population. Our findings highlight a significant knowledge gap in CRCI mitigation research, warranting further larger-scale RCTs employing intervention techniques with a direct influence on the nervous system, such as non-invasive brain stimulation, neurorehabilitation targeting the sensorium, or (electro) acupuncture.

## Data Availability

The original contributions presented in the study are included in the article/**Supplementary Material**. Further inquiries can be directed to the corresponding author.
