# Supplementary material for: Chemotherapy-related cognitive impairment and non-pharmacological interventions targeting the nervous system: a systematic review
Source: Front Psychiatry. 2026 Jun 2;17:1789794. doi: 10.3389/fpsyt.2026.1789794 (PMC13269268; doi:10.3389/fpsyt.2026.1789794)
Supplement: Supplementary file 1 [file DataSheet1.zip › Supplementary Material-Table 1.DOCX]

**Supplementary Table A1. Risk of bias for case-reports**

| **Citation** | demographics | patient history and presented as timeline | current clinical condition | diagnostic tests or assessment methods clearly described | interventions or treatment procedures described | post intervention condition described | adverse events or unanticipated events described | does the case report provide a takeaway lesson |
| --- | --- | --- | --- | --- | --- | --- | --- | --- |
| **Knotkova et al. (2014)** | Y | Y | Y | Y | Y | Y | Y | Y |
| **Nelson and Esty (2016)** | Y | Y | Y | Y | Y | Y | Y | Y |
| **Kuo et al. (2023)** | Y | Y | Y | Y | Y | Y | Y | Y |
| **Li et al. (2025)** | Y | Y | Y | Y | Y | Y | Y | Y |

**Supplementary Table A2. Risk of bias for RCTs**

| **Citation** | True random group assignment | Allocation to treatment group concealed | Treatment group similar at baseline | Participants blind to treatment assignment | Treatment deliverers blind to treatment assignment | Treatment groups identically treated | Outcome assessors blind to treatment assignment | Outcomes measured in same way for treatment groups | Outcomes measured in reliable way | Follow up completed | Participants analyzed in groups they were randomized to | Appropriate statistical analysis | Trial design appropriate |
| --- | --- | --- | --- | --- | --- | --- | --- | --- | --- | --- | --- | --- | --- |
| **Gaynor et al. (2020)** | N/A | N/A | N/A | Y | N | Y | N | Y | Y | Y | U | U | N |
| **Li et al. (2022)** | Y | U | Y | N | N | Y | U | Y | Y | Y | Y | N | Y |
| **Tong et al. (2018)** | Y | U | Y | N | N | Y | Y | Y | Y | U | Y | Y | Y |
| **Zhang et al. (2020)** | Y | Y | Y | Y | N | Y | Y | Y | Y | Y | Y | Y | Y |
| **Rostock et al. (2013)** | Y | Y | U | Np | Np | Y | N/A | Y | Y | Y | Y | Y | Y |
| **Chan et al. (2023)** | Y | U | Y | Y | N | Y | Y | Y | Y | Y | Y | Y | Y |
| **Shen et al. (2025)** | Y | U | Y | N | N | Y | Y | Y | Y | Y | U | Y | Y |
| **Miao et al. (2022)** | Y | U | U | N | N | Y | U | Y | U | Y | U | Y | Y |
| **Du et al. (2021)** | Y | U | N | N | N | Y | N | Y | U | Y | U | Y | Y |

**Supplementary Table A3. Risk of bias for case series**

| **Citation** | Clear inclusion criteria | Standard and reliable measurement of condition | Valid identification of condition | Consecutive inclusion of participants | Complete inclusion of participants | Clear demographics | Clear clinical information | Outcomes reported | Presenting site demographic information | Appropriate statistical analysis |
| --- | --- | --- | --- | --- | --- | --- | --- | --- | --- | --- |
| **Lyu et al. (2022)** | Y | Y | Y | U | U | Y | Y | Y | Y | U |

**Supplementary Table A4. Risk of bias for quasi-experiment studies**

| **Citation** | Clear cause and effect | Control group | Similar comparison participants | Similar treatment | Multiple measurements of outcome | Outcomes measured in same way | Outcomes measured in reliable way | Follow-up completion | Appropriate statistical analysis |
| --- | --- | --- | --- | --- | --- | --- | --- | --- | --- |
| **Zeng et al. (2018)** | Y | Y | U | Y | Y | Y | Y | Y | U |
| **Sawada et al. (2010)** | Y | Y | Y | Y | Y | Y | Y | U | Y |

**Supplementary Table A5. Risk of bias for Cohort studies**

| **Citation** | Groups similar/same population | Exposure measured similarly | Exposure valid/reliable | Confounders identified | Confounding addressed | Outcome absent at baseline | Outcomes valid/reliable | Follow-up sufficient | Follow-up complete/described | Incomplete follow-up addressed | Appropriate statistical analysis |
| --- | --- | --- | --- | --- | --- | --- | --- | --- | --- | --- | --- |
| **Chien et al. (2021)** | N | N | Y | N | N | N | Y | Y | U | N | Y |

Chan, K., Lui, L., Lam, Y., Yu, K., Lau, K., Lai, M., Lau, W., Tai, L., Mak, C., Bian, Z., & Zhong, L. L. (2023). Efficacy and safety of electroacupuncture for oxaliplatin-induced peripheral neuropathy in colorectal cancer patients: a single-blinded, randomized, sham-controlled trial. *Acupunct Med*, *41*(5), 268-283. <https://doi.org/10.1177/09645284221125421>

Chien, A., Yang, C.-C., Chang, S.-C., Jan, Y.-M., Yang, C.-H., & Hsieh, Y.-L. (2021). Ultrasound Acupuncture for Oxaliplatin-induced Peripheral Neuropathy in Patients With Colorectal Cancer: A Pilot Study. *PM&R*, *13*(1), 55-65. <https://doi.org/https://doi.org/10.1002/pmrj.12361>

Du, X.-t., Tian, W.-p., Liu, B., & Li, L.-n. (2021). Prevention and treatment of acupuncture for cancer-related fatigue caused by chemotherapy of intestinal cancer: A randomized controlled trial: 针刺防治肠癌化疗所致癌因性疲乏的随机对照研究. *World Journal of Acupuncture - Moxibustion*, *31*(2), 83-88. <https://doi.org/https://doi.org/10.1016/j.wjam.2020.11.013>

Gaynor, A. M., Pergolizzi, D., Alici, Y., Ryan, E., McNeal, K., Ahles, T. A., & Root, J. C. (2020). Impact of transcranial direct current stimulation on sustained attention in breast cancer survivors: Evidence for feasibility, tolerability, and initial efficacy. *Brain Stimul*, *13*(4), 1108-1116. <https://doi.org/10.1016/j.brs.2020.04.013>

Knotkova, H., Malamud, S. C., & Cruciani, R. A. (2014). Transcranial Direct Current Stimulation (TDCS) Improved Cognitive Outcomes in a Cancer Survivor With Chemotherapy-induced Cognitive Difficulties. *Brain Stimulation*, *7*(5), 767-768. <https://doi.org/10.1016/j.brs.2014.05.007>

Kuo, P. H., Chen, A. Y., Rodriguez, R. J., Stuehm, C., Chalasani, P., Chen, N. K., & Chou, Y. H. (2023). Transcranial Magnetic Stimulation for the Treatment of Chemo Brain. *Sensors 23*(19), 8017. <https://doi.org/10.3390/s23198017>

Li, W., Yang, M., Huang, J., & Zhang, Q. (2025). Long-term electroacupuncture for low anterior resection syndrome in postoperative rectal cancer patients: case reports [Case Report]. *Frontiers in Medicine*, *Volume 12 - 2025*. <https://doi.org/10.3389/fmed.2025.1517325>

Li, Z., Hao, X., Lei, P., Zhou, L., Chen, C., Tan, T., & Yue, L. (2022). Patients With Breast Cancer Receiving Chemotherapy: Effects of Multisensory Stimulation Training on Cognitive Impairment. *Clin J Oncol Nurs*, *26*(1), 71-77. <https://doi.org/10.1188/22.CJON.71-77>

Lyu, Y. R., Lee, H. Y., Park, H. J., Kwon, O. J., Kim, A. R., Jung, I. C., Park, Y. C., Cho, J. H., Kim, J. E., Kim, M., Lee, J. H., & Kim, J. H. (2022). Electroacupuncture for Cancer-Related Cognitive Impairment: A Clinical Feasibility Study. *Integr Cancer Ther*, *21*, 15347354221098983. <https://doi.org/10.1177/15347354221098983>

Miao, X., Wu, H., Liu, Y., Zhang, S., Li, C., & Hao, J. (2022). Clinical Efficacy of Acupuncture on Neoadjuvant Chemotherapy with Capecitabine plus Paclitaxel and Radiotherapy in Progressive Gastric Cancer. *Journal of Oncology*, *2022*(1), 6156585. <https://doi.org/https://doi.org/10.1155/2022/6156585>

Nelson, D. V., & Esty, M. L. (2016). Neurotherapy As a Catalyst in the Treatment of Fatigue in Breast Cancer Survivorship. *Explore (NY)*, *12*(4), 246-249. <https://doi.org/10.1016/j.explore.2016.04.002>

Rostock, M., Jaroslawski, K., Guethlin, C., Ludtke, R., Schroder, S., & Bartsch, H. H. (2013). Chemotherapy-induced peripheral neuropathy in cancer patients: a four-arm randomized trial on the effectiveness of electroacupuncture. *Evid Based Complement Alternat Med*, *2013*, 349653. <https://doi.org/10.1155/2013/349653>

Sawada, N. O., Zago, M. M. F., Galvão, C. M., Cardozo, F. M. C., Zandonai, A. P., Okino, L., & Nicolussi, A. C. (2010). The Outcomes of Visualization and Acupuncture on the Quality of Life of Adult Cancer Patients Receiving Chemotherapy. *Cancer Nursing*, *33*(5). <https://journals.lww.com/cancernursingonline/fulltext/2010/09000/the_outcomes_of_visualization_and_acupuncture_on.13.aspx>

Shen, Q., Deng, D., Li, G., Ruan, J., Shao, X., Wang, P., Li, X., Li, R., Bao, W., Chen, W., & Lu, C. (2025). Electroacupuncture frequency for chemotherapy-induced neuropathy in breast cancer: a randomized controlled trial. *The Oncologist*, *30*(9), oyaf262. <https://doi.org/10.1093/oncolo/oyaf262>

Tong, T., Pei, C., Chen, J., Lv, Q., Zhang, F., & Cheng, Z. (2018). Efficacy of Acupuncture Therapy for Chemotherapy-Related Cognitive Impairment in Breast Cancer Patients. *Med Sci Monit*, *24*, 2919-2927. <https://doi.org/10.12659/MSM.909712>

Zeng, Y., Cheng, A. S. K., Song, T., Sheng, X., Wang, S., Xie, J., & Chan, C. C. H. (2018). Effects of Acupuncture on Cancer-Related Cognitive Impairment in Chinese Gynecological Cancer Patients: A Pilot Cohort Study. *Integr Cancer Ther*, *17*(3), 737-746. <https://doi.org/10.1177/1534735418777109>

Zhang, Z. J., Man, S. C., Yam, L. L., Yiu, C. Y., Leung, R. C., Qin, Z. S., Chan, K. S., Lee, V. H. F., Kwong, A., Yeung, W. F., So, W. K. W., Ho, L. M., & Dong, Y. Y. (2020). Electroacupuncture trigeminal nerve stimulation plus body acupuncture for chemotherapy-induced cognitive impairment in breast cancer patients: An assessor-participant blinded, randomized controlled trial. *Brain Behav Immun*, *88*, 88-96. <https://doi.org/10.1016/j.bbi.2020.04.035>
