# Supplementary material for: Chemotherapy-related cognitive impairment and non-pharmacological interventions targeting the nervous system: a systematic review
Source: Front Psychiatry. 2026 Jun 2;17:1789794. doi: 10.3389/fpsyt.2026.1789794 (PMC13269268; doi:10.3389/fpsyt.2026.1789794)
Supplement: Supplementary file 1 [file DataSheet1.zip › Search Strategy.DOCX]

**Search Executed: March 25, 2026**

**Search Set Added to Existing Covidence Review: Set #29**

**Database: Ovid MEDLINE(R) ALL <1946 to December 31, 2025>**

**Search Strategy:**

**1**  Chemotherapy-Related Cognitive Impairment/ (212)
**2**  ((neoplas$ or cancer$ or malignan$) and (cognit$ adj2 (impair$ or dysfunction$ or declin$ or disorder$ or deteriorat$ or deficit$))).mp. (9741)
**3**  crci.mp. (454)
**4**  (chemo-fog$ or chemofog$ or chemo-brain$ or chemobrain$ or chemotherap$ or chemo-therap$).mp. (676642)
**5**  exp Antineoplastic Agents/ (1356418)
**6**  ((antineoplas$ or anti-neoplas$ or anticancer$ or anti-cancer$ or antitumor$ or anti-tumor$) adj2 (agent$ or drug$ or medicat$)).mp. (493819)
**7**  5 or 6 (1397132)
**8**  exp Cognitive Dysfunction/ (54057)
**9**  ((cognit$ or mental$) adj2 (impair$ or dysfunction$ or declin$ or disorder$ or deteriorat$ or deficit$)).mp. (509301)
**10**  8 or 9 (509428)
**11**  7 and 10 (4671)
**12**  or/1-4,11 (686935)
**13**  Transcranial Magnetic Stimulation/ (18006)
**14**  Transcranial Direct Current Stimulation/ (6831)
**15**  Deep Brain Stimulation/ (13089)
**16**  Vagus Nerve Stimulation/ (3176)
**17**  Electroconvulsive Therapy/ (14998)
**18**  Electroacupuncture/ (5926)
**19**  Acupuncture/ (2136)
**20**  Acupuncture Therapy/ (22230)
**21**  ((transcrani$ or trans-crani$ or brain$ or vagus$ or vagal$ or tragus$ or theta$ burst$ or thetaburst$) adj4 (stimulat$ or photobiomodulat$ or photo-bio-modulat$)).mp. (78773)
**22**  tdcs$.mp. (8824)
**23**  ((sensor$ or cerebral$ or cognit$) adj4 stimulat$).mp. (14605)
**24**  (gamma$ entrain$ or electroacupunc$ or electr$ acupunc$).mp. (9423)
**25**  acupunctur$.ti,kf. (23879)
**26**  or/13-25 (141647)
**27**  12 and 26 (742)
**28**  ..l/ 27 lg=en (663)
**29**  **remove duplicates from 28 (662)**

**Search Executed: March 25, 2026**

**Search Set Added to Existing Covidence Review: Set #32**

**Database: Embase Classic+Embase <1947 to 2025 December 31>**

**Search Strategy:**

**1**  chemotherapy related cognitive impairment/ (519)
**2**  cancer related cognitive impairment/ (336)
**3**  ((neoplas$ or cancer$ or malignan$) and (cognit$ adj2 (impair$ or dysfunction$ or declin$ or disorder$ or deteriorat$ or deficit$ or disturbanc$))).mp. (17064)
**4**  crci.mp. (690)
**5**  (chemo-fog$ or chemofog$ or chemo-brain$ or chemobrain$).mp. (956)
**6**  exp cancer chemotherapy/ (707167)
**7**  ((cancer$ or neoplas$ or malignan$) adj2 (chemotherap$ or chemo-therap$)).ti. (11920)
**8**  exp *antineoplastic agent/ (1635887)
**9**  ((antineoplas$ or anti-neoplas$ or anticancer$ or anti-cancer$ or antitumor$ or anti-tumor$) adj2 (agent$ or drug$ or medicat$)).ti. (31614)
**10**  8 or 9 (1641319)
**11**  exp *cognitive defect/ (436385)
**12**  (cognit$ adj2 (impair$ or dysfunction$ or declin$ or disorder$ or deteriorat$ or deficit$ or defect$ or disturbanc$)).ti. (86746)
**13**  11 or 12 (444827)
**14**  10 and 13 (4233)
**15**  or/1-7,14 (730866)
**16**  exp transcranial magnetic stimulation/ (43192)
**17**  transcranial direct current stimulation/ (17292)
**18**  brain depth stimulation/ (66714)
**19**  vagus nerve stimulation/ (18074)
**20**  electroconvulsive therapy/ (29385)
**21**  electroacupuncture/ (12358)
**22**  *acupuncture/ (34846)
**23**  ((transcrani$ or trans-crani$ or brain$ or vagus$ or vagal$ or tragus$ or theta$ burst$ or thetaburst$) and (stimulat$ or photobiomodulat$ or photo-bio-modulat$)).ti. (61739)
**24**  tdcs$.mp. (14618)
**25**  ((sensor$ or cerebral$ or cognit$) and stimulat$).ti. (10657)
**26**  (gamma$ entrain$ or electroacupunc$ or acupunc$).ti. (38644)
**27**  or/16-26 (221367)
**28**  15 and 27 (972)
**29**  ..l/ 28 lg=en (928)
**30**  remove duplicates from 29 (924)
**31**  (pediatric$ or paediatric$ or infan$ or neonat$ or neo-nat$ or newborn$ or new$ born$ or child$).ti,jx. (2823822)
**32**  **30 not 31 (903)**

**Search Executed: March 25, 2026**

**Search Set Added to Existing Covidence Review:**

**Database: Scopus; to 2025 December 31>**

**Search Strategy:**

( ( TITLE-ABS-KEY ( transcranial AND electric AND stimulation )

OR TITLE-ABS-KEY ( transcranial AND magnetic AND stimulation )

OR TITLE-ABS-KEY ( cranial AND stimulation )

OR TITLE-ABS-KEY ( vagal AND nerve AND stimulation )

OR TITLE-ABS-KEY ( theta AND burst AND stimulation )

OR TITLE-ABS-KEY ( brain AND stimulation )

OR TITLE-ABS-KEY ( electroconvulsive AND therapy )

OR TITLE-ABS-KEY ( transcranial AND direct AND current AND stimulation )

OR TITLE-ABS-KEY ( transcranial AND alternate AND current AND stimulation )

OR TITLE-ABS-KEY ( transcranial AND photobiomodulation )

OR TITLE-ABS-KEY ( electroacupuncture )

OR TITLE-ABS-KEY ( acupuncture ) ) )

AND ( TITLE-ABS-KEY ( cancer ) )

AND ( ( TITLE-ABS-KEY ( chemotherapy AND related AND cognitive AND impairment )

OR TITLE-ABS-KEY ( chemotherapy AND related AND cognitive AND decline )

OR TITLE-ABS-KEY ( chemotherapy AND induced AND cognitive AND impairment )

OR TITLE-ABS-KEY ( chemotherapy AND induced AND cognitive AND decline )

OR TITLE-ABS-KEY ( chemotherapy AND associated AND cognitive AND impairment )

OR TITLE-ABS-KEY ( chemotherapy AND associated AND cognitive AND decline ) ) )

AND PUBYEAR < 2026

**Search Executed: March 25, 2026**

**Search Set Added to Existing Covidence Review:**

**Database: PubMed; to 2025 December 31>**

**Search Strategy:**

(((("transcranial electric stimulation"[Title/Abstract]) OR
("transcranial magnetic stimulation"[Title/Abstract]) OR
("cranial stimulation"[Title/Abstract]) OR
("vagal nerve stimulation"[Title/Abstract]) OR
("theta burst stimulation"[Title/Abstract]) OR
("brain stimulation"[Title/Abstract]) OR
("electroconvulsive therapy"[Title/Abstract]) OR
("transcranial direct current stimulation"[Title/Abstract]) OR
("transcranial alternating current stimulation"[Title/Abstract]) OR
("transcranial photobiomodulation"[Title/Abstract]) OR

("electroacupuncture"[Title/Abstract]) OR
("acupuncture"[Title/Abstract])))
AND ("cancer"[Title/Abstract]))
AND ((("chemotherapy related cognitive impairment"[Title/Abstract]) OR
("chemotherapy related cognitive decline"[Title/Abstract]) OR
("chemotherapy induced cognitive impairment"[Title/Abstract]) OR
("chemotherapy induced cognitive decline"[Title/Abstract]) OR
("chemotherapy associated cognitive impairment"[Title/Abstract]) OR
("chemotherapy associated cognitive decline"[Title/Abstract])))) AND (("1946/01/01"[Date - Publication] : "2025/12/31"[Date - Publication]))

**Search Executed: March 25, 2026**

**Search Set Added to Existing Covidence Review:**

**Database:** PsycINFO**; to 2025 December 31**

**Search Strategy:**

((transcranial electric stimulation

or transcranial magnetic stimulation

or cranial stimulation

or vagal nerve stimulation

or theta burst stimulation

or brain stimulation

or electroconvulsive therapy

or transcranial direct current stimulation

or acupuncture

or electroacupuncture

or transcranial alternating current stimulation

or transcranial photobiomodulation).ti,ab.)

and

(cancer).ti,ab.

and

( chemotherapy related cognitive impairment

or chemotherapy related cognitive decline

or chemotherapy induced cognitive impairment

or chemotherapy induced cognitive decline

or chemotherapy associated cognitive impairment

or chemotherapy associated cognitive decline).ti,ab.

limit to yr="1806 - 2025"**Search Executed: April 7, 2025**

**Search Set Added to Covidence Review: #31**

**Database: Embase Classic+Embase <1947 to 2025 April 04>**

**Search Strategy:**

**1**  chemotherapy related cognitive impairment/ (290)
**2**  cancer related cognitive impairment/ (138)
**3**  ((neoplas$ or cancer$ or malignan$) and (cognit$ adj2 (impair$ or dysfunction$ or declin$ or disorder$ or deteriorat$ or deficit$ or disturbanc$))).mp. (14260)
**4**  crci.mp. (496)
**5**  (chemo-fog$ or chemofog$ or chemo-brain$ or chemobrain$).mp. (782)
**6**  exp cancer chemotherapy/ (662384)
**7**  ((cancer$ or neoplas$ or malignan$) adj2 (chemotherap$ or chemo-therap$)).ti. (11361)
**8**  exp *antineoplastic agent/ (1483107)
**9**  ((antineoplas$ or anti-neoplas$ or anticancer$ or anti-cancer$ or antitumor$ or anti-tumor$) adj2 (agent$ or drug$ or medicat$)).ti. (30208)
**10**  8 or 9 (1488363)
**11**  exp *cognitive defect/ (373385)
**12**  (cognit$ adj2 (impair$ or dysfunction$ or declin$ or disorder$ or deteriorat$ or deficit$ or defect$ or disturbanc$)).ti. (74142)
**13**  11 or 12 (381376)
**14**  10 and 13 (3583)
**15**  or/1-7,14 (682636)
**16**  exp transcranial magnetic stimulation/ (36559)
**17**  transcranial direct current stimulation/ (13531)
**18**  brain depth stimulation/ (60020)
**19**  vagus nerve stimulation/ (15966)
**20**  electroconvulsive therapy/ (27864)
**21**  electroacupuncture/ (10771)
**22**  ((transcrani$ or trans-crani$ or brain$ or vagus$ or vagal$ or tragus$ or theta$ burst$ or thetaburst$) and (stimulat$ or photobiomodulat$ or photo-bio-modulat$)).ti. (53199)
**23**  tdcs$.mp. (11522)
**24**  ((sensor$ or cerebral$ or cognit$) and stimulat$).ti. (9113)
**25**  (gamma$ entrain$ or electroacupunc$ or electr$ acupunc$).ti. (7422)
**26**  or/16-25 (167111)
**27**  15 and 26 (568)
**28**  ..l/ 27 lg=en (543)
**29**  remove duplicates from 28 (541)
**30**  (pediatric$ or paediatric$ or infan$ or neonat$ or neo-nat$ or newborn$ or new$ born$ or child$).ti,jx. (2659047)
**31**  **29 not 30 (528)**

**Search Executed: April 7, 2025**

**Search Set Added to Existing Covidence Review:**

**Database: Scopus; to April 04**

**Search Strategy:**

( ( TITLE-ABS-KEY ( transcranial AND electric AND stimulation )

OR TITLE-ABS-KEY ( transcranial AND magnetic AND stimulation )

OR TITLE-ABS-KEY ( cranial AND stimulation )

OR TITLE-ABS-KEY ( vagal AND nerve AND stimulation )

OR TITLE-ABS-KEY ( theta AND burst AND stimulation )

OR TITLE-ABS-KEY ( brain AND stimulation )

OR TITLE-ABS-KEY ( electroconvulsive AND therapy )

OR TITLE-ABS-KEY ( transcranial AND direct AND current AND stimulation )

OR TITLE-ABS-KEY ( transcranial AND alternate AND current AND stimulation )

OR TITLE-ABS-KEY ( transcranial AND photobiomodulation ) )

AND ( TITLE-ABS-KEY ( cancer ) )

AND ( ( TITLE-ABS-KEY ( chemotherapy AND related AND cognitive AND impairment )

OR TITLE-ABS-KEY ( chemotherapy AND related AND cognitive AND decline )

OR TITLE-ABS-KEY ( chemotherapy AND induced AND cognitive AND impairment )

OR TITLE-ABS-KEY ( chemotherapy AND induced AND cognitive AND decline )

OR TITLE-ABS-KEY ( chemotherapy AND associated AND cognitive AND impairment )

OR TITLE-ABS-KEY ( chemotherapy AND associated AND cognitive AND decline ) ) )

**Search Executed: April 7, 2025**

**Search Set Added to Existing Covidence Review:**

**Database: PubMed; to 2025 April 4**

**Search Strategy:**

(((("transcranial electric stimulation"[Title/Abstract]) OR
("transcranial magnetic stimulation"[Title/Abstract]) OR
("cranial stimulation"[Title/Abstract]) OR
("vagal nerve stimulation"[Title/Abstract]) OR
("theta burst stimulation"[Title/Abstract]) OR
("brain stimulation"[Title/Abstract]) OR
("electroconvulsive therapy"[Title/Abstract]) OR
("transcranial direct current stimulation"[Title/Abstract]) OR
("transcranial alternating current stimulation"[Title/Abstract]) OR
("transcranial photobiomodulation"[Title/Abstract]))
AND ("cancer"[Title/Abstract]))
AND ((("chemotherapy related cognitive impairment"[Title/Abstract]) OR
("chemotherapy related cognitive decline"[Title/Abstract]) OR
("chemotherapy induced cognitive impairment"[Title/Abstract]) OR
("chemotherapy induced cognitive decline"[Title/Abstract]) OR
("chemotherapy associated cognitive impairment"[Title/Abstract]) OR
("chemotherapy associated cognitive decline"[Title/Abstract])))) AND (("1946/01/01"[Date - Publication] : "2025/04/04"[Date - Publication]))

**Search Executed: April 7, 2025**

**Search Set Added to Existing Covidence Review:**

**Database:** PsycINFO**; to 2025 April 4**

**Search Strategy:**

((transcranial electric stimulation

or transcranial magnetic stimulation

or cranial stimulation

or vagal nerve stimulation

or theta burst stimulation

or brain stimulation

or electroconvulsive therapy

or transcranial direct current stimulation

or transcranial alternating current stimulation

or transcranial photobiomodulation).ti,ab.)

and

(cancer).ti,ab.

and

( chemotherapy related cognitive impairment

or chemotherapy related cognitive decline

or chemotherapy induced cognitive impairment

or chemotherapy induced cognitive decline

or chemotherapy associated cognitive impairment

or chemotherapy associated cognitive decline).ti,ab.

limit to yr="1806 - 2025"

**Search Executed: April 3, 2025**

**Search Set Added to Covidence Review: #26**

**Database: Ovid MEDLINE(R) ALL <1946 to April 02, 2025>**

**Search Strategy:**

**1**  Chemotherapy-Related Cognitive Impairment/ (150)
**2**  ((neoplas$ or cancer$ or malignan$) and (cognit$ adj2 (impair$ or dysfunction$ or declin$ or disorder$ or deteriorat$ or deficit$))).mp. (8954)
**3**  crci.mp. (348)
**4**  (chemo-fog$ or chemofog$ or chemo-brain$ or chemobrain$ or chemotherap$ or chemo-therap$).mp. (641430)
**5**  exp Antineoplastic Agents/ (1313576)
**6**  ((antineoplas$ or anti-neoplas$ or anticancer$ or anti-cancer$ or antitumor$ or anti-tumor$) adj2 (agent$ or drug$ or medicat$)).mp. (472322)
**7**  5 or 6 (1352238)
**8**  exp Cognitive Dysfunction/ (46875)
**9**  ((cognit$ or mental$) adj2 (impair$ or dysfunction$ or declin$ or disorder$ or deteriorat$ or deficit$)).mp. (477814)
**10**  8 or 9 (477930)
**11**  7 and 10 (4414)
**12**  or/1-4,11 (651010)
**13**  Transcranial Magnetic Stimulation/ (16697)
**14**  Transcranial Direct Current Stimulation/ (6036)
**15**  Deep Brain Stimulation/ (12344)
**16**  Vagus Nerve Stimulation/ (2808)
**17**  Electroconvulsive Therapy/ (14740)
**18**  Electroacupuncture/ (5540)
**19**  ((transcrani$ or trans-crani$ or brain$ or vagus$ or vagal$ or tragus$ or theta$ burst$ or thetaburst$) adj4 (stimulat$ or photobiomodulat$ or photo-bio-modulat$)).mp. (72875)
**20**  tdcs$.mp. (7870)
**21**  ((sensor$ or cerebral$ or cognit$) adj4 stimulat$).mp. (13662)
**22**  (gamma$ entrain$ or electroacupunc$ or electr$ acupunc$).mp. (8606)
**23**  or/13-22 (107307)
**24**  12 and 23 (317)
**25**  ..l/ 24 lg=en (282)
**26**  **remove duplicates from 25 (282)**
**27**  ("25085269" or "32305573" or "32353419" or "33613570" or "34639353" or "36973688" or "36981862" or "37243381" or "37836847" or "38446899").ui. (10)
**28**  27 not 26 (2)
