# Supplementary material for: Chemotherapy-related cognitive impairment and non-pharmacological interventions targeting the nervous system: a systematic review
Source: Front Psychiatry. 2026 Jun 2;17:1789794. doi: 10.3389/fpsyt.2026.1789794 (PMC13269268; doi:10.3389/fpsyt.2026.1789794)
Supplement: Supplementary file 1 [file DataSheet1.zip › Supplementary Material-Table 2.DOCX]

Supplementary Table 1. Included studies Publication information

| **Citation** | **Authors** | **Publication year** | **Title** | **Study type** |
| --- | --- | --- | --- | --- |
| (Knotkova et al., 2014) | Knotkova,Helena; Malamud,Stephen C;Cruciani,Ricardo A | 2014 | Transcranial Direct Current Stimulation (TDCS) Improved Cognitive Outcomes in a Cancer Survivor With Chemotherapy-induced Cognitive Difficulties | Case report |
| (Nelson et al., 2016) | Nelson,David V;Esty, MaryLee | 2016 | Neurotherapy As a Catalyst in the Treatment of Fatigue in Breast Cancer Survivorship | Case report |
| (Kuo et al., 2023) | Kuo, Phillip, H; Chen, Allison Yu-Chin;Rodriguez,Rudolph J;Stuehm,Carol;Chalasani,Pavani;Chen, Nan-Kuei; Chou,Ying-Hui | 2023 | Transcranial Magnetic Stimulation for the Treatment of Chemo Brain | Case report |
| (Li et al., 2025) | Li W, Yang M, Huang J, Zhang Q | 2025 | Long-term electroacupuncture for low anterior resection syndrome in postoperative rectal cancer patients: case reports | Case report (2 cases) |
| (Lyu et al., 2022) | Lyu, YeeRan; Lee,Hye-Yoon;Park, Hyo-Ju; Kwon,O-Jin; Kim,Ae-Ran;Jung, InChul; Park,Yang-Chun;Cho, Jung-Hyo; Kim,Jung-Eun;Kim,Mikyung;Lee, Jun-Hwan; Kim,Joo-Hee | 2022 | Electroacupuncture for Cancer-Related Cognitive Impairment: A Clinical Feasibility Study | Case series- one arm study |
| (Zeng et al., 2018) | Zeng, Y.;Cheng,A.S.K.;Song, T.;Sheng, X.;Wang, S.;Xie, J.;Chan, C.C.H. | 2018 | Effects of Acupuncture on Cancer-Related Cognitive Impairment in Chinese Gynecological Cancer Patients: A Pilot Cohort Study | Pilot, prospective cohort study  double arm study  Quasi experimental |
| (Sawada et al., 2010) | Sawada NO, Zago MMF, Galvão CM, Cardozo FMC, Zandonai AP, Okino L, Nicolussi AC | 2010 | The Outcomes of Visualization and Acupuncture on the Quality of Life of Adult Cancer Patients Receiving Chemotherapy | Quasi-experimental non-randomized two-group study |
| (Chien et al., 2021) | Chien, A., Yang, C.-C., Chang, S.-C., Jan, Y.-M., Yang, C.-H., & Hsieh, Y.-L | 2021 | Ultrasound Acupuncture for Oxaliplatin-induced Peripheral Neuropathy in Patients With Colorectal Cancer: A Pilot Study | Prospective pilot cohort study |
| (Gaynor et al., 2020) | Gaynor,AlexandraM; ;Pergolizzi,Denise;Alici, Yesne;Ryan,Elizabeth;McNeal,Katrazyna;Ahles, TimA; Root,James | 2020 | Impact of transcranial direct current stimulation on sustained attention in breast cancer survivors: Evidence for feasibility, tolerability, and initial efficacy. | Crossover design |
| (Li et al., 2022) | Li, Zeyuan; ;Hao, Xijun;Lei, Ping;Zhou, Lizhi;Chen,Changxiang;Tan,Tingting;Yue, Liming | 2022 | Patients With Breast Cancer Receiving Chemotherapy: Effects of Multisensory Stimulation Training on Cognitive Impairment. | Randomized controlled trial |
| (Tong et al., 2018) | Tong, T.;Pei, C.;Chen, J.; Lv,Q.; Zhang,F.; Cheng, Z. | 2018 | Efficacy of Acupuncture Therapy for Chemotherapy-Related Cognitive Impairment in Breast Cancer Patients | Blinded randomized controlled trial |
| (Zhang et al., 2020) | Zhang,Zhang-Jin; ;Man, Sui-Cheung;Yam, Lo-Lo;Yiu, ChuiYing; Leung,RolandChing-Yu;Qin, Zong-Shi; Chan,Kit-WaSherry; Lee,Victor HoFun; Kwong,Ava; Yeung,Wing-Fai;So, WinnieK W; Ho, LaiMing; Dong,Ying-Ying | 2020 | Electroacupuncture trigeminal nerve stimulation plus body acupuncture for chemotherapy-induced cognitive impairment in breast cancer patients: An assessor-participant blinded, randomized controlled trial. | Assessor-participant blinded, randomized controlled trial |
| (Rostock et al., 2013) | Rostock, M;;Jaroslawski,K; Guethlin,C; Ludtke,R; Schroder,S; Bartsch,H H | 2013 | Chemotherapy-induced peripheral neuropathy in cancer patients: a four-arm randomized trial on the effectiveness of electroacupuncture. | randomized, placebo-controlled trial  four arm study |
| (Chan et al., 2023) | Kaiyin Chan, Louisa Lui, Yukting Lam, Kaling Yu, Kwongwai Lau, Manchi Lai, Waiwai Lau, Lokyin Tai, Chunkin Mak, Zhaoxing Bian and Linda LD Zhong | 2023 | Efficacy and safety of electroacupuncture for oxaliplatin-induced peripheral neuropathy in colorectal cancer patients: a single-blinded, randomized, sham-controlled trial | single blinded randomized sham-controlled trial |
| (Miao et al., 2022) | Miao X, Wu H, Liu Y, Zhang S, Li C, Hao J | 2022 | Clinical Efficacy of Acupuncture on Neoadjuvant Chemotherapy with Capecitabine plus Paclitaxel and Radiotherapy in Progressive Gastric Cancer | Randomized controlled clinical trial |
| (Shen et al., 2025) | Shen Q, Deng D, Li G, Ruan J, Shao X, Wang P, Li X, Li R, Bao W, Chen W, Lu C | 2025 | Electroacupuncture frequency for chemotherapy-induced neuropathy in breast cancer: a randomized controlled trial | Single-center, single-blind randomized controlled trial |
| (Du et al., 2021) | Du XT, Tian WP, Liu B, Li LN | 2021 | Prevention and treatment of acupuncture for cancer-related fatigue caused by chemotherapy of intestinal cancer: A randomized controlled trial | Randomized controlled trial |

Supplementary Table 2. Included studies timeframe and reported location

| **Citation** | **Location** | **Recruitment period** | **Intervention period** | **Follow-up period** |
| --- | --- | --- | --- | --- |
| (Knotkova et al., 2014) | Not reported | Not reported | 5 days | 2 weeks |
| (Nelson et al., 2016) | Not reported | Not reported | 10 weeks and an additional session 1 month later | 6 months |
| (Kuo et al., 2023) | University of Arizona  AZ; USA | Not reported | 2 weeks | No follow-up |
| (Li et al., 2025) | Beijing University of Chinese Medicine, China | Not reported | 6 months | 6 months |
| (Lyu et al., 2022) | Daejeon and Dunsan Korean Medicine Hospital of Daejeon University, | 2016.11.23.-2018.01.31. | 8 weeks | 4 weeks |
| (Zeng et al., 2018) | The Hong Kong Polytechnic University  The Third Affiliated Hospital of Guangzhou Medical University | Not reported | 5 weeks  average 10 sessions (2x a week) | No follow-up |
| (Sawada et al., 2010) | Specialized Oncology Center in Ribeirao Preto, Sao Paulo, Brazil. | 2007.01. – 2008.12. | Weekly sessions during 6-month chemotherapy protocol; on average ~24 sessions | End of chemotherapy (~6 months from baseline); no long-term follow-up |
| (Chien et al., 2021) | Cheng Ching General Hospital and China Medical University Hospital | Not reported | Baseline phase: 12 days Intervention phase: 12 days | Withdrawal phase: 30 days - after this they have the follow-up visit on day 54 compared to the start of the study |
| (Gaynor et al., 2020) | Memorial Sloan Kettering Cancer Center  NY; USA | 2017.05. -2019.07. | Participants completed four visits over four consecutive days. 2 days of active stimulation and 2 days of sham stimulation during cognitive task.  4 days total | No Follow-up, |
| (Li et al., 2022) | Tangshan  People’s Hospital, Hebie China | 2018.03- 2019.04. | 26 days, delivered 4 times, total 96 days | 4 rounds of the intervention, without any further specification on follow-up |
| (Tong et al., 2018) | China Affiliated Hospital of Jiangnan University China | 2017.05. -2017.10. | Patients received two 4-week courses of acupuncture with a 3-day rest between the 2 courses. Every week, intervention was delivered once a day for 5 days, followed by 2 days of rest. | No follow-up: assessment was done right after the last session. Patients lost to follow-up is referring to patients lost through the treatment period. |
| (Zhang et al., 2020) | School of Chinese Medicine of the University of Hong Kong. | 2015.10.- 2018 12. | 8 weeks long intervention | Not reported |
| (Rostock et al., 2013) | Tumor Biology Center at the Albert Ludwigs University Freiburg, Germany. | 2000.09.-2003.02. | 21 days | They applied for a 63-day long follow-up, with follow-up visit on 84th days from baseline. |
| (Chan et al., 2023) | Yan Chai Hospital-Hong Kong Baptist University Chinese Medicine Clinic cum Training and Research Centre (Kwai Tsing District) (KTCMC). | 2018.08.-2020.01. | 12 weeks (weekly sessions) | 12 weeks follow-up |
| (Miao et al., 2022) | Changzhou Central Hospital, China | 2018.05.– 2020.06. | Acupuncture delivered during chemoradiotherapy; treatment course aligned with neoadjuvant protocol | No long-term follow-up |
| (Shen et al., 2025) | Zhejiang Cancer Hospital, China | 2022.01.-2023.10. | 12 sessions over 4 weeks | 4-week post-intervention follow-up |
| (Du et al., 2021) | Guangdong Provincial Hospital of Chinese Medicine, China | 2018.06. –2019.01. | 2 chemotherapy cycles; 8 acupuncture treatments total | 3 weeks after the second chemotherapy cycle |

Supplementary Table 3. Included studies Participants description

| **Citation** | **Patient demographics (n, age, sex)** | **Cancer type** | **Cancer stage and additional reporting** | **Chemotherapy regime** |
| --- | --- | --- | --- | --- |
| (Knotkova et al., 2014) | 1 patient  55 years old Female | Breast Cancer | Not reported | Trastuzumab: docetaxel and carboplatin for 18 weeks, then Trastuzumab alone for an additional 34 weeks. |
| (Nelson et al., 2016) | 1 patient 45 years old Female | Breast cancer | Not reported | Not reported |
| (Kuo et al., 2023) | 1 patient 58 years old Female | Breast cancer | grade III invasive ductal carcinoma with one out of four sentinel lymph nodes positive for cancer. Hormone receptor positive and human epidermal growth factor receptor 2 negative | 4 cycles of adjuvant chemotherapy:  Docetaxel; Cyclophosphamide; Tamoxifen; Anastrozole; Letrozole |
| (Li et al., 2025) | n=2; Case 1 male age 66; Case 2 male age 65 | Rectal cancer | Case 1: pT3N2M0; Case 2: pT3N0M0 | CAPOX regimen- 2 cycle of -oxaliplatin 200 mg IV on day 1; Capecitabine 1.5 g orally twice daily on days 1–14, followed by a 7‑day long break,  Case 1: neoadjuvant CAPOX ×2 then adjuvant oxaliplatin + capecitabine ×6; with lowered oxaliplatin dosage (180 mg) Case 2: adjuvant capecitabine ×8; Capecitabine 1.25 g orally twice daily on days 1–14 of each 21‑day cycle, followed by a 7‑day drug‑free interval |
| (Lyu et al., 2022) | 12 patients Average age 57.33 years old 2 males and 10 females. | 8 were diagnosed with breast cancer,  2 with thyroid cancer,  1 with colon cancer,   1 with kidney cancer, | Varied stages from stage Ⅰ to Ⅳ.   All 12 subjects had undergone surgery for cancer treatment, 9 had received chemotherapy, 3 received radiation therapy, and 6 received either hormonal therapy or targeted therapy. | No further reporting |
| (Zeng et al., 2018) | 15 patients (of those, 3 cognitive impaired) 49.33 ± 9.14 (28-60) all female.  15 control -age matched 49.60 ± 8.27 (29-59) all female | Cervical cancer 8 (53.3) Ovarian cancer 1 (6.7) Uterine cancer 6 (40.0) | Early stage (stage I-IIa) 9 (60.0) Middle stage (stage IIb-IIIa) 3 (20.0) Advanced stage (stage IIIb) 3 (20.0) | Surgery + chemotherapy 13 (86.7) Surgery + chemotherapy + radiation 2 (13.3) |
| (Sawada et al., 2010) | n=75 final sample;   intervention: n=38; 24 (63.2%) female, 14 (36.8%) male ; Age, y (n(%)) : 20-40Y: 3 (7.9%); 40-60Y:21 (55.3%); 60-80Y: 14 (36.8%)  control: n=37; 24 (64.9%) female, 13 (35.1%) male; Age, y (n(%)): 20-40Y: 6 (16.2%); 40-60Y: 14 (37.9% ) 60-80Y: 17 (45.9% ) | Intervention (n=38) Gastrointestinal cancer 17 (44.7%)  Gynecologic & breast cancer \| 12 (31.6%)  Prostate cancer 1 (2.6%)  Metastasis (21.1%)  Surgery 36 (94.7%)  Radiotherapy 18 (47.4%)  Chemotherapy Sessions  6 sessions 35 (92.1%) ≥6 sessions 3 (7.9%)  Control (n=37) : Gastrointestinal cancer 16 (43.3%) Gynecologic & breast cancer 15 (40.5%)  Prostate cancer 3 (8.1%)  Metastasis 3 (8.1%)  Surgery 35 (94.6%) Radiotherapy 9 (24.3%) Chemotherapy Sessions 6 sessions 22 (59.5%) ≥6 sessions11 (29.7%) | Not reported | Chemotherapy: Regimen varied and were not fully specified |
| (Chien et al., 2021) | n=17; mean age 65.6 ± 10.1 years; 7 male, 10 female | Colorectal cancer | Stage II–IV (inclusion) stage III -88.2% (15/17)   stage IV 11.8% (2/17) | Oxaliplatin-based regimens: fluorouracil, leucovorin, and oxaliplatin   FOLFOX4, oxaliplatin dose of 85 mg/m2- (n=10 58.8%) FOLFOX6, oxaliplatin dose of 100 mg/m2 (n=7 41.2%) capecitabine plus oxaliplatin (XELOX;oxaliplatin dose of 130 mg/m2 Average time since initiation: 2.1 ± 1.1 years Range of time since initiation: 8 months to 4 years Years since cessation of chemotherapy <1 y 2 11.8%; 1–3 y 12 70.6%, >3 y 3 17.6% |
| (Gaynor et al., 2020) | 16 patients. Age between 45-60.  All females. | Breast cancer | Not reported | Unknown chemotherapies |
| (Li et al., 2022) | 80 patients between 18-60 years old ;all female   Multisensory group: 40 Audiovisual group: 40 | Breast Cancer | Multisensory TNM: I 18; II 18; III 4  Audiovisual TNM: I 18; II 17; III 5 | Two chemotherapy cycles, with the total number of prescribed cycles  being 6 or more of the ACT (epirubicin, cyclophosphamide, and  docetaxel) chemotherapy regimen |
| (Tong et al., 2018) | 75 patients. Treatment group (39)  mean age 43.11 ± 4.23   Control group:  mean age 42.26± 4.42  No specification on gender, based on the design presumably all women | Breast cancer | Not reported | Total chemotherapy duration was 3–6 months. Supportive medications included antacids (lansoprazole, omeprazole), antiemetics (ondansetron, palonosetron), and dexamethasone for allergy prevention. Granulocyte colony-stimulating factor was administered when complications such as anemia or bone marrow suppression occurred.  TC: docetaxel 75 mg/m² + cyclophosphamide 600 mg/m² IV on day 1, every 21 days for 4 cycles.  TCb: docetaxel 75 mg/m² + carboplatin (AUC 6) IV on day 1, every 21 days for 4 cycles.  AC→T: doxorubicin 60 mg/m² + cyclophosphamide 600 mg/m² IV on day 1, every 21 days for 4 cycles, followed by docetaxel 100 mg/m² IV on day 1, every 21 days for 4 cycles. |
| (Zhang et al., 2020) | 92 patients Average age:47.9 All female.  EA/TNS + BA: (n = 45) years:47.9 ± 9.7  MAS  (n = 47) years: 47.9 ± 10.6 | Breast cancer | EA/TNS + BA : I: 6 (13.3%); II: 25 (55.6%); IIIa: 14 (31.1%) MAS : I: 10 (21.3%); II: 25 (53.2%); IIIa: 12 (25.5%) p = 0.574 | Under or post chemotherapy at entry, n (%) EA/TNS + BA: Under: 33 (73.3%); Post: 12 (26.7%) MAS: Under: 37 (78.7%); Post: 10 (21.3%) p = 0.718 Chemotherapies: FEC-T regimen: EA/TNS+BA 9 (20.0 %); MAS 11 (23.4%) TAC regimen: EA/TNS+BA 7 (15.6 %); MAS 7 (14.9%) TC regimen: EA/TNS+BA 12 (26.7 %); MAS 9 (19.0%) AC regimen: EA/TNS+BA 2 (4.4%), MAS 0 TPH regimen: EA/TNS+BA 11 (24.4 %), MAS 15 (31.9%) Others: EA/TNS+BA 4 (8.9 %), MAS 5 (10.6%) |
| (Rostock et al., 2013) | 60 patients  EA:  mean age 49,9( SD: 9.6) 10 female , 4 male   HB: mean age 52.3 (SD:11.3) 12 female, 1 male   VitB:  mean age 56.3 (SD: 11.1) 10 female, 5 male  Placebo :  mean age 52.0 (SD:8.1) 14 female, 3 male | EA:  Breast cancer 6 (42.9%)  Ovarian Cancer 3 (21.4%) Lymphoma 4 (28.6%)  Other 1 (7.1%)  secondary cancer 2 (14.3%)  HB: Breast cancer 3 (23.1%)  Ovarian Cancer 3 (23.1%) Lymphoma 5 (38.5%)  Other 2 (15.4%)  secondary cancer 3 (23.1.%)  VitB:  Breast cancer 4 (26.7%)  Ovarian Cancer 4 (26.7%) Lymphoma 6 (40.0 %)  Other 1 (6.7%)  secondary cancer 4 (26.7%)  Placebo :  Breast cancer 8 (47.1%)  Ovarian Cancer 3 (17.6%) Lymphoma 2 (11.8%)  Other 4 (7.1%)  secondary cancer 2 (23.5%) | Not reported | EA:  Vinca alkaloids 4 (28.6%) Platin derivatives alone 1 (7.1%) Taxanes alone 6 (42.9%) Platin derivatives and taxanes combined 3 (21.4%) Total no. of different cytostatics 2.1 ± 1.4 No. of diff. neurotoxic cytostatics only 1.6 ± 1.2   HB: Vinca alkaloids 5 (28.5%) Platin derivatives alone 2 (15.4%) Taxanes alone 3 (23.1%) Platin derivatives and taxanes combined 3 (23.1%) Total no. of different cytostatics 1.5 ± 0.9 No. of diff. neurotoxic cytostatics only 1.1 ± 0.3   VitB Vinca alkaloids 6 (40.0%) Platin derivatives alone 0 (0%) Taxanes alone 4 (26.7%) Platin derivatives and taxanes combined 5 (33.3%) Total no. of different cytostatics 1.7 ± 1.0 No. of diff. neurotoxic cytostatics only 1.2 ± 0.4    Placebo Vinca alkaloids 3 (17.6) Platin derivatives alone 3 (17.6%) Taxanes alone 8 (47.1%) Platin derivatives and taxanes combined 5 (29.4%) Total no. of different cytostatics 2.0 ± 0.8 No. of diff. neurotoxic cytostatics only 1.3 ± 0.6 |
| (Chan et al., 2023) | Male 18 (33%) (EA) 15 (27%) (S) Female EA:9 (16%); Sham 13 (24%)  Age 60.0 ± 8.57 (EA) 62.5 ± 7.62 0.308 (S)  Gender p=0.322 | Colorectal carcinoma | Electroacupuncture group (n = 27):  Stage III: 24 (44%) Stage IV: 3 (6%)  Sham acupuncture group (n = 28):  Stage III: 24 (44%) Stage IV: 4 (7%) | Chemotherapy regimen p= 0.669 XELOX 24 (44%) (EA) 26 (47%) (S) FOLFOX or other 3 (6%) (EA) 2 (4%) (S) |
| (Miao et al., 2022) | n=70; intervention n=35 aged 46–64, control n=35 aged 41–63; mean age 52.17 ± 4.38 vs 52.34 ± 5.14; intervention 17 male/18 female, control 20 male/15 female | Advanced gastric cancer | Control: 35 Stage III  Acupuncture:35 Stage III | Capecitabine 1,650 mg/(m²·d) orally d1–d14 + paclitaxel 175 mg/(m²·d) IV d1; 2 cycles; radiotherapy 45 Gy/25 fractions |
| (Shen et al., 2025) | n=152 MITT population; mean age 53.30 ± 8.94 years; female breast cancer patients  2 Hz EA (n=39) age: 53.74 (8.44) 100 Hz EA (n=38) age: 54.39 (8.25) 2/100 Hz EA (n=37) age : 52.02 (9.62) Mecbl (n=38) age: 53.02 (9.61) | Breast cancer with chemotherapy-induced peripheral neuropathy | Not reported | Taxane-containing chemotherapy (nab-paclitaxel, paclitaxel liposome, paclitaxel, docetaxel; alone or in combination) |
| (Du et al., 2021) | n=61 enrolled;   treatment group: 26 ; 18 male, 8 female; mean age 55.62 ± 12.04    control: 24;15 male and 9 female; 61.83 ± 10.55;   Pearson chi-square test (χ2) showed that there was no significant difference in sex between the two groups (χ2 = 0.252, p = 0.616, > 0.05. | Colorectal cancer | Not reported | FOLFOX,OR FOLFIRI, OR XELOX regimens 2 cycles ( 6 weeks total)   Patients received chemotherapy administered according to NCCN (2018) guidelines and clinical practice; FOLFOX included oxaliplatin (130 mg/m²), leucovorin (400 mg/m²), and fluorouracil (2800 mg/m²) every 3 weeks,  FOLFIRI included irinotecan (180 mg/m²), leucovorin (400 mg/m²), and fluorouracil (2800 mg/m²) every 3 weeks,  and XELOX consisted of oxaliplatin (130 mg/m²) and capecitabine (1000 mg/m²) every 3 weeks. |

Supplementary Table 4. Included studies Intervention protocols

| **Citation** | **Intervention type** | **Intervention protocol and control group protocol** |
| --- | --- | --- |
| (Knotkova et al., 2014) | transcranial Direct Current Stimulation | Intervention group:  Baseline assessment, followed by 5 sessions of tDCS on 5 consecutive days and a follow-up at two weeks after tDCS completion. tDCS intensity 2 mA -20 min/session - 5 session on consecutive days . "1*1 Transcranial Direct Current Low-Intensity Stimulator" (model 1300 A(Soretix Medical)). Two saline-soaked sponge ( 4.5 * 6 cm), the anode placed over the left dorsolateral prefrontal cortex, point F3 of the international EEG 10-20 classification, and the cathode over the contralateral supraorbital region  Control group protocol:  NA |
| (Nelson et al., 2016) | Flexyx Neurotherapy System (FNS) | Intervention group:  At a time, pre-set in the software, the dominant (peak) EEG frequency had an additional +20 Hz of electromagnetic (EM) stimulation applied for a maximum one-second burst of alternating on/off pulsing. For example, if the momentary dominant frequency was 10 Hz, the EM stimulation frequency for a maximum one-second pulsing was 30 Hz. Hence, the dose of EM stimulation was directly dependent on the momentary peak frequency in the EEG at the time the system was pre-set to administer the stimulation. The first intervention session included one second of stimulation for each of three sites. The number of sites treated in each session then varied from two to three, depending on the effects and any side effects, proceeding through the sequence of sites determined by the initial assessment. If bothersome discomfort between sessions was reported, the therapist had the option to vary the number of sites, including holding off entirely. Patients subsequently attended 10 weekly sessions with stimulation and an 11th one month later.  Control group protocol:  NA |
| (Kuo et al., 2023) | Transcranial Magnetic Stimulation | Intervention group:  The intermittent theta burst stimulation (iTBS) protocol was delivered using a MagVenture MagPro X100 stimulator (MagVenture Inc., Farum, Denmark) equipped with figure-of-eight magnetic coils (MagVenture C-B60 and Cool-B65 coils, MagVenture Inc., Farum, Denmark). iTBS pulses were administered for a total of 600 pulses within a 192-second session. The protocol comprised repeated triplet bursts at 50 Hz delivered in 2-second “on” periods (30 pulses), followed by 8-second “off” periods with no stimulation. 10 consecutive sessions of intermittent theta burst stimulation were administered during weekdays over the course of two weeks.  Control group protocol:  NA |
| (Li et al., 2025) | Electroacupuncture | Intervention group:  Disposable sterile acupuncture needles (0.30 x 75 mm) and an SDZ-V Hwato-brand electroacupuncture device were used. Stimulation acupoints were the following-baliao points- bilateral Shangliao (BL31), Ciliao (BL32), Zhongliao (BL33), and Xialiao (BL34), plus Changqiang - GV1; with 50 Hz continuous wave; 30 min/session; twice weekly for 6 months. Control group:  NA |
| (Lyu et al., 2022) | Electroacupuncture | Intervention group: A total of 16 electroacupuncture treatments, twice a week for 30 minutes for 8 weeks. Acupuncture points:   GV20, GV24, and EX-HN1, and bilateral: GB20, HT7, PC6, and KI3.  Participants were then treated with an electro stimulator (ES-160, Ito Co. Ltd., Tokyo, Japan) at the acupoint of both sides HT7 and PC6 at a frequency of 2 Hz and 80% intensity for 30 minutes.  Control group:  Study was conducted as single arm study, without a control group. |
| (Zeng et al., 2018) | Acupuncture | Intervention group:  Patients with cognitive impairment at the time of diagnosis were invited to receive manual acupuncture, which was provided by a single acupuncturist trained in traditional Chinese medicine.  Sterile, disposable, stainless steel needles (0.25 mm in diameter and 40 mm in length, Huanqiu brand, made in China) were inserted at the following forehead acupuncture points: EX-HN1 (left and right, anterior and posterior Sishencong), EX-HN3 (Yintang), EX-HN5 (bilateral Taiyang), GB8 (bilateral Shuaigu), GB15 (Toulinqi), GB20 (Fengchi), GV20 (Baihui), ST8 (bilateral Touwei),  unilaterally or bilaterally, depending on each woman’s traditional diagnosis (constitution) as determined by the acupuncturist.  The duration of needling was 30 minutes, and the frequency of interventions was 2 times per week.  As the total number of chemotherapy cycles for gynecological cancer patients ranged from 4 to 6 cycles, the average total number of interventions was 10 sessions per patient.  The depth of needling varied between 25 and 40 mm, depending on the individual point.  Control group:  Age and disease stage matched patient controls -NO acupuncture intervention, only receiving routine/usual care. |
| (Sawada et al., 2010) | Acupuncture plus relaxation with visualization | Weekly 15-minute relaxation with visualization before chemotherapy infusion plus 20-minute acupuncture using standardized symptom-directed points during chemotherapy.  Relaxation with Visualization was conducted before chemotherapy infusion, led by trained nurse researchers and included the following: Progressive body relaxation, Visualization of disease and immune response, Visualization of healing and recovery, Mental imagery of nature and desired activities,  Positive reinforcement and emotional engagement,  Acupuncture Protocol Delivered by a medical doctor specialized in acupuncture. Needles: Disposable (0.25 × 40 mm) Frequency: Once weekly for 6 months; Duration: ~20 minutes per session The acupuncture protocol included both bilateral and unilateral acupoints. The bilateral acupoints comprised PC6 (Neiguan), ST36/ E 36 (Zusanli), SP6 (Sanyinjiao), and LR2 (Xingjian). In addition, several unilateral midline acupoints were used, including CV12/RM 12 (Zhongwan), CV17/RM17 (Shanzhong/Tanzhong), and EX-HN3 (Yintang).  Control group:  Standard chemotherapy only |
| (Chien et al., 2021) | Ultrasound acupuncture | Pulsed ultrasound (1 MHz, 50% duty cycle), ultrasound output gradually increased to elicit a deqi sensation than 5 min per point daily for 12 days. Ultrasound device- (US-750, ITO Co, Tokyo, Japan.) had a 1.7-cm-diameter treatment head and an effective radiating area of 0.75 cm2.  Bilateral application to the following acupoints were the following, upper extremities: PC6 (Neiguan) and PC7 (Daling); lower extremities: BL60 (Kunlun) and KI1 (Yongquan). Intervention was delivered while receiving their respective oxaliplatin-based treatment regimens.  Control group was missing, one arm design. |
| (Gaynor et al., 2020) | Transcranial Direct Current Stimulation | Intervention group (cross over design- every participant received both active and sham intervention): Intervention was 30 min long. Starstim wireless hybrid EEG/tES multichannel transcranial current stimulator (http://www.neuroelectrics.com) was used to administer tDCS stimulation. Two Ag/AgCl electrodes with a 1cm radius were used to administer stimulation over the left dorsolateral prefrontal cortex (dlPFC): the anode (stimulating electrode) was placed over the F3 position, and cathode (return electrode) was placed over F4 based on the 10–20 EEG system  Active sessions: tDCS device delivered a ramp-down stimulation over 30 s and the experimental run of the computerized CPT task was initiated. Once the ramp-up was complete, a steady state current of 1 mA was administered for the duration of the behavioral task (15 min). Sham sessions: Stimulation was ramped up over 30 s and then down over 30 s at both the start and end of the task, with no active current delivered through the duration of the task. |
| (Li et al., 2022) | audiovisual and multisensory by random sampling | Within this design the audiovisual group is considered as control group, and the active/intervention group received the same audiovisual training and additional stimulating. First, we present the control/ audiovisual group protocol followed by the additions incorporated into the multisensory group’s protocol.  One treatment cycle lasted for 26 days, consisting of 5 days of hospitalization for routine treatment and 21 days of rest at home. The stimulation training lasted for 20 minutes each day. The cycles of treatment were repeated four times.  Audiovisual training (Based on the 66nao Brain Training system developed by Wispirit Tech) was comprised of three modules: photo–name matching, listen and link, and sequence memory. The photo–name matching module was designed to train patients’ ability to recognize faces, match photos and names, and improve delayed recall memory. The listen and link module trained patients’ semantic processing ability and working memory. Finally, the sequence memory module focused on training patients’ memory and execution capability. Researchers could log into the system and check patients’ daily training and the duration of patients being hospitalized. The multisensory group received audiovisual intervention and scalp tactile stimulation and tactile training with objects. The scalp tactile stimulation involved three steps. First, participants were instructed to keep their fingers adjusted together and massage the temples in the clockwise and anticlockwise direction. Second, press the ring finger to the forehead area from the glabella to the Baihui acupoint at the top of the head, then along the midline and continuing downward to the Fengchi acupoint at the nape. Third, fingertips on both hands were used to gently comb the head, starting at the forehead and moving toward the back of the head. Tactile stimulation, objects were placed in a cardboard box to feel size/ temperature/ texture using both hands., the audiovisual training was comprised of three modules: photo–name matching, listen and link, and sequence memory. The photo–name matching module was designed to train patients’ ability to recognize faces, match photos and names, and improve delayed recall memory. The listen and link module trained patients’ semantic processing ability and working memory. Finally, the sequence memory module focused on training patients’ memory and execution capability. The multisensory group also participated in an intervention method that comprised olfactory memory stimulation training and olfactory stimulation training during bedtime.  The olfactory memory stimulation training involved the nurses preparing two test strips infused with different essential oils. One test strip was placed under the nose for 20 seconds, so the patient could memorize the scent. After two minutes, the participant smelled the other test strip. After five minutes, the patient was asked to name the two essential oils in order. During bedtime, the olfactory stimulation training comprised of nurses lighting a lavender-scented candle and placing it beside the participant’s bed while relaxing music was played. Patients engaged in diaphragmatic breathing for 10 minutes. The tactile and olfactory stimulation training were performed once a day for five consecutive days, lasting for 10 minutes each time. These cycles were repeated four times. |
| (Tong et al., 2018) | Acupuncture | Basic acupuncture formulas Baihui (DU20), Sishencong (EX-HN1), and Taixi (KI3) were used.  Based on symptoms and tongue manifestation, other acupoints could also be stimulated as follows. The angle insertion of Baihui, Shenting (DU24), and Sishencong are approximately 10–20° (between needle and scalp), with 10–15 mm as the best insertion depth. Taixi, Dazhong (KI4), and Juegu (GB39) were inserted 15–20 mm deep with a 0.25×25 mm acupuncture needle. Zusanli (ST36) was inserted 25–35 mm deep with a 0.25×40 mm acupuncture needle.  Effective needling was accompanied by needling feelings of numbness, tingling, swelling, or muscle weakness, known as “de qi” in acupuncture; the needle was kept in situ for 30 min after stimulation.  Sterile, disposable needles 40 mm long and 0.25 mm in diameter (Huatuo, Suzhou Medical Instruments Factory, China) were used by acupuncturists. Data collection: before acupuncture therapy (time 1) and after completion of acupuncture therapy (time 2). The control group completed assessments at the same time points.  Control group: not receiving acupuncture, without further specification |
| (Zhang et al., 2020) | Eelectroacupuncture trigeminal nerve stimulation plus body acupuncture (EA/TNS + BA)   Minimum acupuncture stimulation (MAS) | Intervention group: For EA/TNS + BA**:** manual stimulation of:  Bilateral -Shenmen (HT7), Hegu (LI4), Waiguan (TE5), Zusanli (ST36), Fenglong (ST40) and Sanyinjiao (SP6) Midline: Zhongwan (CV12), Guanyuan (CV4), and Shuigou (GV26)  **Electrical stimulation:** with positive (+) and negative (−) electrode cord connection:  Baihui (GV20, +) and Yintang (EX-HN3,−); **Left :**Sishencong (EX-HN1, −) and Toulinqi (GB15, +),  **Right:** Sishencong (EX-HN1, −) and Toulinqi (GB15, +), **Bilateral:** Shuaigu (GB8, L+, R−), Taiyang (EX-HN5, L+, R−), Touwei (ST8, L+, R−). The peak current and voltage of the machine (model: ITO ES-360) were 6 V and 48 mA, respectively, with constant wave at frequency of 2 Hz and phase duration of 100 μs for 30 min.  Control group- MAS: The following 6 acupoints were used; bilateral Tongtian (BL7, L+, R−),  bilateral Shousanli (LI10), bilateral Fuyang (BL59). Electrical stimulation was only performed on bilateral Tongtian (BL17) Parameters are the same as within the active group (EA/TNS+BA), but the intensity was adjusted to a level at which patients just started feeling stimulation. |
| (Rostock et al., 2013) | Electroacupuncture (EA)   Hydroelectric Baths (HB).   Vitamin B Complex (Vit B)   Placebo | Intervention group:  Patients with CIPN symptoms in the upper and lower extremities were treated with the complete point combinatio, needles were deeply inserted bilaterally until the deqi phenomenon (sensation which spreads over the whole-body part described as “aching,” “soreness,” “pressure,” or “tingling” was triggered. Each session included 15 minutes of electrostimulation (50Hz) consisting of a combination of rectangular currents and high amplitude waves. The stimulus strength increased until the deqi phenomenon was triggered again.  Electroacupuncture was employed to effected extremities, be the following point combination: LV3 (Taichong), SP9 (Xiongxiang), GB41 (Zulingqi), GB34 (Yanglingquan) (legs; in patientswithCIPN symptoms in the lower extremities) and LI4 (Hegu), LI11 (Quchi), SI3 (Houxi), and HT3 (Shaohai) (arms; in patients with CIPN symptoms in the upper extremities). 8 ± 1 sessions of EA were administered.  Control groups: Hydroelectric Baths (HB) 8 ± 1 session were scheduled to treat the affected extremities. The patients dipped their arms up to a hand’s width above the elbow and their feet up to a hand’s width above the ankle into a special water basin with water at a temperature of about 35∘C. The water served as an electrode for the skin surface. Each treatment lasted for 15 minutes with cross-galvanization of each individual extremity by low-frequency (50Hz) faradic current (direct current impulses) up to the individual’s sensitive threshold (i.e., the point where the tingling feeling is just acceptable).  Vitamin B Complex (Vit B): The treatment consisted of 3 capsules of high-dosage vitamin B1/B6 (100mg thiamine nitrate, 100mg pyridoxine hydrochloride) per day for three weeks.  Placebo: treatment consisted of 3 lactose capsules per day identical in form, taste, and odor to theVitB capsules. |
| (Chan et al., 2023) | Electro acupucnture (EA)  vs Sham (SA) | Intervention group:  The EA intervention was performed weekly (one session per week) over 12 consecutive weeks  acupuncture point locations for treatment of CIPN were Bafeng, Baxie, LR3 (Taichung) and LI4 (Hegu).24 With the clinical experience of our PI and Co-Is, the following eight traditional acupuncture point locations were chosen: LI4, PC6 (Neiguan), LI11 (Quchi), Baxie, ST36 (Zusanli), SP6 (Sanyinjiao), LR3 and Bafeng. The use of Baxie and Bafeng was optional if skin lesions of the hands and feet occurred due to Capecitabine (Xeloda). Registered Chinese Medicine practitioner with more than 5 years of Chinese medicine college education plus at least 5 years of clinical experience.  Disposable stainless steel acupuncture needles (0.25 × 25 mm Hwato verum acupuncture needles matching the Streiterger sham needles) were inserted to a depth of 10–25 mm at the sites. Electrical stimulation was delivered with continuous waves at 2 Hz frequency (pulse width 200 ms) and a variable intensity titrated to each patient’s minimum sensation of stimulation (range 2–5 mA) using an EA instrument (KWD808I multipurpose health device, Ying Di, Chang Zhou, China). The needles were retained for 25 min.  Control group:  For patients assigned to the SA group Streitberger’s non-invasive acupuncture needles (gauge 8 × 1.2″/0.30 × 30 mm) were applied to serve as sham control at the same point locations (LI4(Hegu),  PC6 (Neiguan), LI11 (Quchi), Baxie, ST36 (Zusanli), SP6 (Sanyinjiao), LR3 and Bafeng.) Same perceived stimulation modality, except that the needles were only adhered to the skin by a small plastic ring instead of being inserted and the EA was a form of ‘pseudostimulation’, achieved by connecting the needle to the incorrect output socket of the EA instrument. |
| (Miao et al., 2022) | Acupuncture plus chemoradiotherapy | Intervention group: 20-minute long acupuncture sessions were delivered with additional moxibustion at CV4(Guanyuan), CV6 (Qihai) , ST36 (Zusanli), SP15 (Daheng), PC6 (Neiguan), SP10 (Xuehai), SP8 (Diji), ST28 (Shuidao), and ST29 (Guilai) during conventional treatment (Lifting-thrusting method and Reinforcing-reducing method ). A ~2 cm moxa stick was placed on the needle handle 2–3 cm above the skin, ignited, and allowed to burn completely during a ~20-minute session, providing gentle heat at the acupoint. Kraft paper protected the skin, and cardboard could be used to reduce heat if needed.  Control group:  Patients received routine care consisting of neoadjuvant chemotherapy with capecitabine and paclitaxel combined with radiotherapy, followed by regular clinical monitoring, including blood tests and assessment of liver and kidney function. Nutritional support was provided throughout treatment. After completion of chemotherapy, gastroscopy and CT imaging were performed to evaluate treatment response, and patients subsequently underwent surgical resection (radical gastrectomy with D2 lymph node dissection) within a few weeks. |
| (Shen et al., 2025) | Electroacupuncture (2 Hz, 100 Hz, or 2/100 Hz) vs mecobalamin  Eligible patients were randomized in a 1:1:1:1 ratio to receive 2 Hz EA, 100 Hz EA, 2/100 Hz EA, or mecobalamin (MeCbl) | Intervention group:  EA groups received 12 sessions, 30 min/session, every other day (3/week) for 4 weeks at standardized upper/lower limb acupoints; comparator received oral mecobalamin for 4 weeks Iintensity :0.5–4 mA, adjusted to patient tolerance to produce a clear sensation without discomfort.  Patients were followed up for 4 weeks after the final intervention. Upper limbs were treated bilaterally at LI11 (Quchi), SJ5 (Waiguan), LI4 (Hegu), SI3 (Houxi), and EX-UE9 (Baxie), with electroacupuncture (EA) connected between LI4 (Hegu) and SJ5 (Waiguan). Lower limb symptoms, bilateral acupoints included GB34 (Yanglingquan), ST36 (Zusanli), SP9 (Yinlingquan), SP6 (Sanyinjiao), LR3 (Taichong), and EX-LE10 (Bafeng), with EA applied between ST36 (Zusanli) and SP6 (Sanyinjiao). Patients with symptoms in all limbs received both upper and lower limb protocols. EA was delivered at frequencies of 2 Hz, 100 Hz, or alternating 2/100 Hz, depending on group allocation. Treatment utilized Huatuo disposable needles (0.25 × 40 mm) and a HANS-200E acupoint nerve stimulator. Control group:  Patients in the MeCbl group received treatment with MeCbl tablets orally (produced by Misato Plant of Eisai Co., Ltd). They were instructed to take 0.5 mg at a time, three times daily, a total of 1.5 mg a day for 4 consecutive weeks. And were also followed up for 4 weeks after the last intervention. |
| (Du et al., 2021) | Acupuncture plus chemotherapy | Intervention group:  Reinforcing acupuncture at CV6, CV4, and bilateral ST36;  Needles were inserted slowly in the direction of the meridian with minimal rotation, followed by gentle manipulation after achieving deqi. The technique involved light rotation, heavy thrusting with gentle lifting, and low-frequency, small-amplitude movements from superficial to deeper layers. Needles were retained for 30 minutes, with manipulation performed after insertion and before removal, followed by rapid pressing and kneading of the insertion site. Acupuncture was administered during two chemotherapy cycles, with four sessions per cycle (one day before chemotherapy and on days 1, 2, and 3), totaling two treatment courses. Frequency of intervention-30 min/session; 1 day before chemotherapy and on days 1–3 of each chemotherapy cycle; 4 sessions/cycle for 2 cycles  Control group:  Chemotherapy was given for two cycles, 6 weeks in total |

Supplementary Table 5. Applied cognitive measurements and impact on cognition

| **Citation** | **Assessed cognitive domain** | **Intervention related findings** |
| --- | --- | --- |
| (Knotkova et al., 2014) | Neurotax- validated computerized testing battery Cognitive Outcomes:  Memory score: assessed using the Immediate Recognition and Delayed Recognition tests.  Executive function score: evaluated using the Go/No-Go Test, Stroop Interference Test, and Catch Game.  Attention score: measured by response times in the Go/No-Go and Stroop Interference tests.  Global cognitive score: calculated from performance across all three cognitive domains (memory, executive function, and attention).  Self-reported outcomes:  Quality of Life Enjoyment and Satisfaction Questionnaire  Patient Global Impression of Change Scale (PGIC)  Patient Satisfaction Rating Scale  Symptom assessment:  Brain fog symptoms were rated using an 11-point numerical rating scale. | The Global cognitive score: before 88.7(below average.),> 108.6, (normal) >2w follow-up (103.9).  The total Memory score: 79.4, (> 1 SD below average) > 114.9 and 111.9 respectively.  The score of Executive Function: 89.5 > 108.2> 101.3   Attention was in average range: 97.2, suggesting that this cognitive domain was not affected by the chemo-fog. (97.2> 102.8> 98.5, ) Patient’s Global Impression of Change scale after the last tDCS session and at follow up was 6 and 5-6 out of 7 respectively. Ratings of brain-fog symptoms: 6 out of 10 before tDCS to 0.7 at the follow-up visit.  The Quality of Life score increased from 50 > 60 at the follow-up,  Hamilton Depression Rating Scale : 14 > 11. |
| (Nelson et al., 2016) | Cognitive clouding  Revised Piper Fatigue Scale and subscale scores:  (Total; Behavioral/Severity; Affective meaning; Sensory; Cognitive/Mood) Center for Epidemiologic Studies-Depression (CES-D) | Current Symptom ratings: Fatigue: Pre-treatment:7; Post-treatment:2  Cognitive clouding: Pre-treatment: 5; Post-treatment:3 Pain: Pre-treatment: 5; Post-treatment:3 Sleep quality: Pre-treatment:6; Post-treatment:3  Anxiety: Pre-treatment:6; Post-treatment:2  Depression: Pre-treatment:4; Post-treatment:2 Irritability/anger: Pre-treatment: 4; Post-treatment:2  Overall activity: Pre-treatment:4; Post-treatment:2  Current symptom0–10 ratings collected at beginning of treatment session; not collected at six-month follow-up. |
| (Kuo et al., 2023) | Working Memory: Forward Digit Span; Backward Digit Span  Verbal Memory: Rey Auditory Verbal Learning Test   Semantic Memory: Verbal Fluency Task  Cognitive Flexibility/Response Inhibition: Stroop Color-Word Interference Task | Working Memory : Forward Digit Span : 7 >8; Backward Digit Span :8> 8  Verbal Memory : RAVLT **: 49 >66  Semantic Memory : Verbal Fluency Task: 33> 32 Cognitive Flexibility/Response Inhibition: Stroop Color-Word Interference Task 33 > 36 |
| (Li et al., 2025) | EORTC QLQ-C30 cognitive function | Both cases showed improved cognitive function scores alongside major bowel symptoms and QoL improvement.  Case 1’s cognitive score improved from 33 to 100; Case 2 from 17 to 50. |
| (Lyu et al., 2022) | Primary outcome: subjective measurement -FACT-Cog(V3); perceived cognitive impairments (PCI);  perceived cognitive abilities (PCA); impact on quality of life (IQL); comments from others (CFO)  Secondary outcomes:  Korean version of the Montreal Cognitive Assessment (MoCA-K),  - objective cognitive function and measuring -memory, language, executive functions, visuospatial skills, calculation, abstraction, attention, concentration, and orientation. Seoul Neuropsychological Screening Battery (SNSB),  evaluating cognitive functioning : attention, language and related functions, visuospatial functions, memory, frontal/executive functions, and other indexes  Boston naming test-verbal memory (BNT) European Organization for Research and Treatment of Cancer Quality of Life Core Questionnaire C30 (EORTCQLQ- C30); physical function, role, emotion, cognition, and social function | BNT: 51.7 → 53.90 (w8, p=0.0207) → 54.90 (w12, p=0.0003) FACT-Cog: 81.17 → 93.70 (w4, p=.145) → 90.04 (w8, p=.326); Δ=+12.54 (w4), +19.98 (w12) PCI: 45.67 → 59.33 (w12, p=0.0227); PCA: 12.75 → 14.56 (p=0.531); CFO: 12.75 → 14.14 (p=0.307); IQL: 10.00 → 13.11 (p=0.115) MoCA-K: 25.50 → 27.73 (w4, p=0.0007) → 28.40 (w8, p=0.0002) → 28.90 (w12, p=0.0004) Visuospatial/executive: 4.25 → 4.90 (w12, p=0.0368); Abstraction: peak ↑ at w8 (p=0.0368) SNSB-II: baseline mild visuospatial & memory decline → significant ↑ in memory, language (+ attention, visuospatial, executive). SVLT & RCFT: immediate/delayed recall + recognition ↑ (significant at w8 & w12) |
| (Zeng et al., 2018) | Learning & memory: AVLT-R (Chinese version of Auditory Verbal Learning Test–Revised) Information processing speed: TMT-A (Trail Making Test A) Executive function: TMT-B (Trail Making Test B) Attention & working memory: WAIS-III Digit Span Verbal fluency & language: COWA (Controlled Oral Word Association Test) | Post-intervention, cognitive test scores were comparable between the intervention (n=3) and cancer control (n=3) groups across all domains. For attention and working memory, Digit Span Forward scores were 6.76 (1.94) versus 6.57 (2.87), and Digit Span Backward scores were 2.11 (1.41) versus 1.85 (1.57). For verbal memory, AVLT immediate recall was 16.65 (6.45) versus 16.28 (3.65), delayed recall 5.86 (1.73) versus 5.42 (3.64), and recognition 10.53 (2.98) versus 10.78 (1.96). Psychomotor speed (TMT-A) was 57.13 (27.48) in the intervention group and 53.80 (21.86) in controls, while executive function (TMT-B) was 75.33 (36.07) versus 74.17 (29.55). Language performance (COWA) was 27.42 (6.89) in the intervention group and 26.76 (9.48) in controls. |
| (Sawada et al., 2010) | EORTC QLQ-C30 cognitive function | Cognitive scores showed no significant change in either group, with the intervention group increasing from 72.18 ± 30.18 to 74.36 ± 27.15 (p = 0.626) and the control group decreasing from 80.40 ± 35.58 to 76.35 ± 27.55 (p = 0.355). Overall, no significant cognitive improvement was observed; however, other quality-of-life domains improved in the intervention group. |
| (Chien et al., 2021) | EORTC QLQ-C30 cognitive functioning | Cognitive functioning showed no significant improvement despite numerical increases over time: 83.33 ± 27.22 → 85.00 ± 24.15 → 91.67 ± 16.20 → 96.67 ± 7.03 (p = .447). Overall, the intervention did not demonstrate a significant effect on cognition. |
| (Gaynor et al., 2020) | Conners’ Continuous Performance Test (CPT-II) were applied to examine attention. (Reaction time and accuracy)  Patient Assessment of Own Functioning Inventory (PAOFI), self-reported -measure of difficulty with memory, attention, concentration, language, and thinking abilities; Sensory Gating Inventory (SGI)-attention and concentration | Self-perceived cognitive improvement (PAOFI) decreased from 97.21 (SD=25.54) to 93.93 (SD=21.90). SGI scores showed a marginally significant reduction from pre-stimulation (M=62.14, SD=30.49) to post-stimulation (M=56.43, SD=29.22; F[1,13]=3.17, p=0.098). For attentional variability, stimulation condition significantly predicted overall Hit RT SE (F[1,38.887]=4.95, p<0.05), with lower values during active stimulation (M=1.546, SE=0.063) compared to sham (M=1.648, SE=0.063), indicating improved response consistency. It was marginally significant for Hit SE ISI Change (F[1,40.16]=3.92, p=0.055). Stimulation significantly predicted Hit RT ISI Change (F[1,38.92]=9.14, p<0.01), with lower values during active stimulation (M=0.044, SE=0.01) versus sham (M=0.057, SE=0.01), suggesting reduced variability in reaction times across ISIs. No significant effect was found for Hit RT Block Change (F[1,42.54]=0.31, p=0.58). At the 4-second ISI, SE was lower during active stimulation (M=2.023, SE=0.083) than sham (M=2.161, SE=0.083), indicating reduced reaction time variability under increased attentional demand. |
| (Li et al., 2022) | Memory , cognitive deficit and executive function  The CRCI and executive functioning of the participants in both groups were assessed before the stimulation training and after four cycles of stimulation training, using the second edition of the Rivermead Behavioural Memory Test (RBMT-II) and the Behavioural Assessment of the Dysexecutive Syndrome (BADS). RBMT II 12 subsets remembering hidden belongings, and picture recognition. Total scores range 0 to 24, with lower scores indicating worse executive function BADS consists of six subtests, including rule-shift cards, action program, and temporal judgment (0 to 4) [0 to 24] lower scores indicating more flawed executive function. | After four intervention cycles, the multisensory group scored significantly higher than the audiovisual group on 11 subtests and the total RBMT-II score (all p<0.05).  Within the audiovisual group, eight subtests and the total RBMT-II score improved significantly post-intervention, while in the multisensory group, all RBMT-II scores showed significant improvement.  The multisensory group also achieved significantly higher scores on four BADS subtests, and the total BADS score compared to the audiovisual group (p<0.001). In both groups, post-intervention BADS scores (four subtests and total) were significantly higher than pre-intervention (audiovisual: p<0.01; multisensory: p<0.0011). RBMT-II assesses memory (e.g., hidden belongings, picture recognition), with total scores ranging from 0–24 (lower scores indicate poorer memory). BADS evaluates executive function across six subtests (e.g., rule-shift cards, action program, temporal judgment), also scored 0–24, with lower scores indicating greater impairment. |
| (Tong et al., 2018) | Self-report measures were:  health information and medical history and  functional assessment of cancer treatment cognition (FACT-COG, version 3).  Neuropsychological tests included: (1) Auditory-Verbal Learning Test (AVLT) for memory—short-term (AVLT1), delayed recall (AVLT2), and recognition (AVLT3); (2) Verbal Fluency Test (VFT; “animals/min”) assessing language, semantic memory, and executive function; (3) Symbol Digit Modalities Test (SDMT) measuring attention, processing speed, and visual working memory; (4) Clock-Drawing Test (CDT) for visual screening of mild-to-moderate cognitive impairment; and (5) Trail-Making Test Part B (TMT-B) assessing executive function and driving-related abilities.  All participants also completed the Mini-Mental State Examination (MMSE), State Anxiety Inventory (S-AI), and Beck Depression Inventory (BDI). | The treatment group had significantly higher scores after acupuncture therapy on FACT-COG, AVLT3, and CDT compared with baseline (paired t test, P<0.05). In contrast, the control group showed no significant differences in performance at time 2 compared to baseline. FACT-COG treatment T1 98.75±12.94 T2 102.38±13.78 T value 4.840** Control T1 99.60±11.05 T2 99.80±10.77 T 1.489 F 5.77 P 0.001 PCI treatment T1 55.42±10.95 T256.29±11.49 T 3.494** Control T1 57.55±8.43 T2 57.35±8.99 T 0.721 F 3.21 P 0.027 PCA treatment T1 20.38±4.19 T2 21.79±4.40 T 2.298* Control T1 19.25±3.31 T2 19.60±3.33 T 1.285 F 3.75 P 0.014 AVLT3 treatment T1 10.92±1.44 T2 11.42±1.18 T 2.202*control T1 10.75±1.59 T2 10.70±1.49 T 0.357 F 5.21 P 0.002 CDT treatment T1 8.08±1.50 T2 8.54±1.14 T 2.696* control T1 8.10±1.21 T2 8.05±1.36 T 0.438 F 5.50 P 0.002 |
| (Zhang et al., 2020) | The primary outcome was cognitive function measured by the Montreal Cognitive Assessment (MoCA). Secondary outcomes included forward and reverse digit span tests to assess attention and working memory. Quality of life was evaluated using EORTC QLQ-C30 and BR23. Functional changes and side effects associated with therapies were measured using the Functional Assessment of Cancer Therapy-Breast Cancer (FACT-B), Functional Assessment of Chronic Illness Therapy (FACIT), Functional Assessment of Anorexia/Cachexia Therapy (FAACT), Functional Assessment of Cancer Therapy-Taxane (FACT-Taxane), and Functional Assessment of Cancer Therapy-Biologic Response Modifier (FACT-BRM) | A linear mixed-effects model showed no significant group × time interaction for MoCA, forward digit span, or reverse digit span (F=0.55, p=0.702; F=0.68, p=0.604; F=1.35, p=0.251, respectively). Significant time effects were observed across all measures (F≥4.98, p≤0.0007). A significant group effect was found only for reverse digit span (F=7.03, p=0.009), with the EA/TNS + BA group scoring higher than MAS at Week 2 (p=0.045) and Week 8. No significant baseline differences between groups across all measures (MoCA, forward, reverse digit span) → supports no group × time interaction finding. Strong time effects in both groups: MoCA and forward digit span improved significantly over time in both groups (all p<0.001 from Week 2 onward). Critical finding (reverse digit span): Between-group differences become significant at: Week 2 (p=0.045); Week 8 (p=0.004). EA/TNS + BA shows consistently larger improvements than MAS. Forward digit span & MoCA: Improvements occur, but no significant between-group differences at any time point. |
| (Rostock et al., 2013) | Quality of life: EORTC QLQ-C30  CIPN symptom severity (primary outcome): Numerical Rating Scale (NRS, 0–10), assessing overall neuropathy burden CIPN symptoms (patient-reported): Numbness, swelling, tingling, pain, and functional impairment (daily life/work), rated by intensity Neuropathy (clinical assessment): Composite neuropathy score (sensory symptoms, pin sensibility, vibration, strength, reflexes; 0–15) Nerve function: Electroneurography (median and sural nerve conduction studies) Neuropathy severity classification: NCI Common Toxicity Criteria (CTC) | EORTC day: 0 EA: mean: 66.7 SD 23.6 /HB mean:39.7 SD 30.1/ VtB mean: 62.2 SD: 28.5 / placebo mean :67.6 SD: 24.6/ Sum: mean: 59.9 SD 28.2 day : 21 >EA: mean: 70.2 SD 18.7/ HB: mean: 55.1 SD 30.0 / VtB : mean 71.1 SD 24.0 / placebo: mean : 79.4 SD 18.2 / Sum: mean: 69.8 SD 23.9 day 84 > EA: mean: 76.7 SD 21.8/ HB: mean: 58.5 SD 36.2/ VtB: mean: 61.6 SD 25.8 / placebo: mean: 71.0 SD13.2 / Sum: mean :67.2 SD 25.2   The neuropathy score decreased in all groups during treatment to a similar degree. Improvements were observed most frequently in the EA group and were smallest in the HB and placebo groups (Table 5). Group differences were not significant between any two groups; for example, the difference between EA and placebo was 𝑑 = −0.4 (CI: −1.1 to 0.3; *p*= 0.128). There were no statistically significant differences between the treatment groups by electroneurographic test results |
| (Chan et al., 2023) | Neuropathy (primary outcome): FACT/GOG-Ntx (11-item subscale; sensory, motor, auditory, dysfunction) Neuropathy symptom severity: Numerical Rating Scale (NRS; 0–10 for numbness/pain) Quality of life: EORTC QLQ-C30 (functional + symptom scales) Vibration sense: Rydel-Seiffer tuning fork test Light touch sensation: 10 g monofilament test (Neuropen) Safety/adverse events: Clinical reports + laboratory tests (CBC, renal, liver function) | Cognitive function **1st cycle:**  **EA**: Mean ± SD 85.2 ± 16.23 change Mean ± SD : 0  **SA** Mean ± SD : 86.3 ± 16.39 ; Change Mean ± SD: –0.60 (–2.00 to 0) ; **Between-group p value :** p= 0.396  **2nd cycle : EA:** Mean ± SD 91.4 ± 13.37 change Mean ± SD : 6.17 (0.52 to 12.34); **SA** Mean ± SD : 88.1 ± 16.27; Change Mean ± SD: 1.19 (–4.76 to 7.05); **Between-group p value :** p= 0.228  **3rd cycle**  **EA:** Mean ± SD 88.3 ± 12.07; change Mean ± SD : 3.09 (–2.47 to 9.44)  **SA** Mean ± SD : 89.3 ± 12.18; Change Mean ± SD: 2.38 (–3.84 to 9.44) ;**Between-group p value :**p=0.565  **Week 12**  **EA**: Mean ± SD 87.7 ± 13.55; change Mean ± SD : 2.47 (–3.70 to 8.33);  **SA** Mean ± SD : 88.1 ± 14.24; Change Mean ± SD: 1.19 (–5.73 to 8.03); **Between-group p value :** p=0.957  **Week 24 EA:** Mean ± SD 77.8 ± 24.02; change Mean ± SD : –7.41 (–18.23 to 3.34)  **SA** Mean ± SD :83.9 ± 17.26; Change Mean ± SD:–2.98 (–8.98 to 2.78); **Between-group p value :** p=0.233   In addition to at baseline, the FACT/GOG-Ntx (11-item) subscale, NRS, vibration sense test and light touch test were assessed every week and the QLQ-C30 was administered every 3 weeks.  The FACT/GOG-Ntx (11 items) subscale score showed a significant difference between the two groups in the second cycle (p = 0.027; Table 2), indicating that the EA group had fewer neuropathy symptoms than the SA group. However, the change in the FACT/GOC-Ntx subscale score over cycles was similar in the two groups. Furthermore, there was no statistically significant difference between the two groups at weeks 12 and 24. |
| (Miao et al., 2022) | Post-treatment quality-of-life cognitive function domain | Cognitive function scores were higher in the acupuncture group than in the control group after treatment.  Cognitive function 78.45 ± 5.85 vs 61.34 ± 6.27; p<0.001. |
| (Shen et al., 2025) | EORTC QLQ-C30 cognitive function; EORTC QLQ-CIPN20 indirect QoL domains | No significant between-group difference in cognitive function; study mainly showed frequency-specific effects on neuropathy and some QoL domains. Baseline cognitive function around 66.67 (IQR 16.67) across groups; no significant cognitive differences at week 4 or week 8. Cognitive function QLQ C30 base lien:  2 Hz EA 66.67 (16.67) ; 100HzEA 66.67 (16.67); 2/100 HzEA- 66.67 (16.67); Mecbl 75.00 (16.67)  Cognitive function at week 4  2 Hz EA 0.0 (0.0) p=0.14; 100HzEA 0.0 (0.0) p=0.89; 2/100 HzEA 0.0 (16.67) p=0.10; Mecbl 0.0 (0.0) p=0.93 Cognitive function at week 8  2 Hz EA 0.0 (8.33) p=0.43; 100HzEA 0.0 (16.67) p=0.10; 2/100 HzEA 0.0 (16.67) p=0.14; Mecbl 75.00 (16.67) 0.0 (0.0) p=0.79 |
| (Du et al., 2021) | PFS cognition dimension; EORTC QLQ-C30 cognitive function | PFS cognition improved significantly in the acupuncture group, whereas QLQ-C30 cognitive function did not show significant change. PFS cognition ([Mean ± SD /M(Q)].) treatment group: 4.61 ± 1.43 to 3.86 ± 1.15 (p<0.05) in comparison before treatment within the group;  control 3.65 ± 1.92 to 3.81 ± 1.83 (NS).  QLQ-C30 cognitive ([Mean ± SD /M(Q)].) Treatment group: 66.67 (20.84) to 69.23 (16.79) (NS).;  Control: 66.67 (33.33) to 63.89 ± (26.77) (NS). |

Supplementary Table 6. Weaknesses of design and limitation

| **Citation** | **Weakness of design/ Authors reported limitation** |
| --- | --- |
| (Knotkova et al., 2014) | Results were derived from results obtained in one patient and the procedure was delivered in an open-label setting. Based on the putative mechanisms underlying tDCS effects, it can be speculated that neuroplastic changes in neural circuits associated with cognitive functions contributed at least partially to the observed functional improvement. |
| (Nelson et al., 2016) | No control, no blinding and no comparison |
| (Kuo et al., 2023) | No control; no blinding; no comparison  A key limitation of this study is the absence of a sham control and its non-blinded design, which may allow for the influence of placebo effects on short-term cognitive enhancement and activity in specific brain regions, such as the dorsolateral prefrontal cortex and the anterior cingulate cortex. |
| (Li et al., 2025) | Two-case report without control or statistical testing.  Lack of control group; possible bias; follow-up descriptive only.  Case report design limits generalizability and causal inference. |
| (Lyu et al., 2022) | Premature termination due to recruitment difficulty; 12 participants enrolled over ~9 months. Heterogeneity in cancer type, stage, and treatment (majority breast cancer); future trials should focus on specific cancer types, stages, and chemotherapy regimens to better assess effect size and mechanisms. Outcome measures should be expanded to better capture intervention effects, including biomarkers and neuroimaging. Although FACT-Cog is widely used, the PCI subscale may be more sensitive than the total score and could serve as a secondary outcome. Balanced use of neuropsychological tests and self-reports is essential. While SNSB was used, ICCTF recommends inclusion of Hopkins Verbal Learning Test–Revised, Trail Making Test, and Controlled Oral Word Association Test. Given SNSB length (>1 hr), shorter versions may improve feasibility and compliance. |
| (Zeng et al., 2018) | small sample size, small number of MCI/ CRCI in the end they compared the 3 CRCI to 3 cancers + non CRCI patients how do not got acupuncture. |
| (Sawada et al., 2010) | The study was limited by a non-randomized design with self-selection into groups and the use of combined interventions, introducing self-selection bias and preventing attribution of effects to acupuncture alone. Additionally, the small sample size and higher dropout rate in the control group further limit the reliability and generalizability of the findings. |
| (Chien et al., 2021) | The study was a single-arm pilot with a small sample size, lacking a sham or control group, resulting in an uncontrolled design with short follow-up. Additionally, heterogeneity in chemotherapy regimens and the fact that cognition was not a primary endpoint further limit the interpretability of the findings. |
| (Gaynor et al., 2020) | One limitation to the design of the current experiment is that for participants who received two days of active stimulation followed by two days of sham, there is a potential for after-effects of active stimulation to persist into the sham days.  limited sample size per group (N=8), and a future randomized controlled trial could separately examine differences in consecutive days of active tDCS compared to sham sessions, |
| (Li et al., 2022) | The sample included only 80 patients with breast cancer aged 18–60 years who were hospitalized for chemotherapy, and those receiving chemotherapy in outpatient clinics were not included. Therefore, generalizability is limited. Because older adults in China have limited use of smartphones, restrictions were placed on the age of the target population. As the effectiveness of this intervention reveals, future research by the study team will also include outpatients receiving chemotherapy, and the age range will be extended to increase the sample size and verify the current findings regarding multisensory stimulation training. |
| (Tong et al., 2018) | There are some limitations to our research. First, the small sample size is not sufficient to provide strong support to the conclusion and further studies are warranted in multicenter, large sample trials. Second, patients willing to participate in studies like this tend to be positive, and thus participants may represent a biased sample. Third, subjective and memory-learning effects occur when using cognitive function scales, and more convincing results can be achieved if combined with neuroimaging technology. In addition, there was no follow-up observation |
| (Zhang et al., 2020) | The study failed to detect a statistically significant difference in MoCA and prevalence of chemobrain between the two groups. MoCA is unable to detect subtle cognitive deterioration- floor effect! (prolonged treatment duration could produce significant differences for MoCA) Assessors and participants were blinded to treatment, however acupuncturists were not blinded to treatment. Bias from acupuncturists could not be completely excluded. Determination of body acupoints used in this study was basically based on empirical evidence. Empirical treatment regimens have resulted in a large variation in acupuncture protocols and difficulties in comparing treatment outcomes among trials. Finally, cognitive performance was measured only with MoCA and digit span test in the current study. Other scales, such as auditory verbal learning test, shape trails test, animal fluency test, Boston naming test, should be included to examine other cognitive domains in the future. In addition, no biomedical approaches, such as neuroimaging or brain function, were included in the measurement of treatment outcomes. |
| (Rostock et al., 2013) | Several limitations should be considered when interpreting the findings. First, the baseline intensity of CIPN symptoms was relatively low, particularly in the electroacupuncture group. As the study design and sample size calculations were based on populations with more severe symptoms and higher pain levels, this likely reduced the potential for measurable improvement and introduced a possible floor effect. Second, the short treatment and observation period of three weeks, dictated by the structure of the rehabilitation program, may have been insufficient given the typically chronic course of CIPN; longer treatment durations—up to 10 weeks in prior studies—may be necessary to observe meaningful effects of acupuncture-based interventions. Third, the study was conducted within a comprehensive rehabilitation setting, where participants received multiple concurrent medical and psychosocial interventions tailored to individual needs. These co-interventions, including physical therapy, psychoeducation, and relaxation techniques, may have contributed to improvements across all groups, including the placebo group, thereby attenuating between-group differences. Finally, the pragmatic nature of the study, with patients recruited from across Germany and treated within routine care, may limit the ability to isolate the specific effects of the interventions under investigation. |
| (Chan et al., 2023) | Key limitations include the small sample size and short intervention and follow-up periods, which may have limited the detection of meaningful effects in chronic CIPN. Longer treatment durations and extended follow-up (up to 12 months) are needed in future studies. The single-blind design introduces potential performance bias, and the lack of assessment of blinding success further limits internal validity. In addition, limited adverse event reporting restricts safety conclusions. Finally, although proposed mechanisms of electroacupuncture are supported by prior research, they were not directly assessed in this study. |
| (Miao et al., 2022) | Small, single-center trial with cognition measured only as a QoL domain. No long-term follow-up or survival statistics. Limited outcome scope; cognition not tested objectively. |
| (Shen et al., 2025) | Pilot/parameter-finding trial; cognition secondary and subjective. Single-center design; no sham EA group; outcomes mostly patient-reported.  Small per-group sample for frequency comparison; acupuncturists unblinded. |
| (Du et al., 2021) | Small, non-blinded RCT with mixed chemotherapy regimens and indirect cognition emphasis. |

References

Chan, K., Lui, L., Lam, Y., Yu, K., Lau, K., Lai, M., et al. (2023). Efficacy and safety of electroacupuncture for oxaliplatin-induced peripheral neuropathy in colorectal cancer patients: A single-blinded, randomized, sham-controlled trial. *Acupunct Med* 41**,** 268-283.

Chien, A., Yang, C.-C., Chang, S.-C., Jan, Y.-M., Yang, C.-H., and Hsieh, Y.-L. (2021). Ultrasound acupuncture for oxaliplatin-induced peripheral neuropathy in patients with colorectal cancer: A pilot study. *PM&R* 13**,** 55-65.

Du, X.-T., Tian, W.-P., Liu, B., and Li, L.-N. (2021). Prevention and treatment of acupuncture for cancer-related fatigue caused by chemotherapy of intestinal cancer: A randomized controlled trial: 针刺防治肠癌化疗所致癌因性疲乏的随机对照研究. *World Journal of Acupuncture - Moxibustion* 31**,** 83-88.

Gaynor, A. M., Pergolizzi, D., Alici, Y., Ryan, E., Mcneal, K., Ahles, T. A., et al. (2020). Impact of transcranial direct current stimulation on sustained attention in breast cancer survivors: Evidence for feasibility, tolerability, and initial efficacy. *Brain Stimul* 13**,** 1108-1116.

Knotkova, H., Malamud, S. C., and Cruciani, R. A. (2014). Transcranial direct current stimulation (tdcs) improved cognitive outcomes in a cancer survivor with chemotherapy-induced cognitive difficulties. *Brain Stimulation* 7**,** 767-768.

Kuo, P. H., Chen, A. Y., Rodriguez, R. J., Stuehm, C., Chalasani, P., Chen, N. K., et al. (2023). Transcranial magnetic stimulation for the treatment of chemo brain. *Sensors* 23**,** 8017.

Li, W., Yang, M., Huang, J., and Zhang, Q. (2025). Long-term electroacupuncture for low anterior resection syndrome in postoperative rectal cancer patients: Case reports. *Frontiers in Medicine* Volume 12 - 2025.

Li, Z., Hao, X., Lei, P., Zhou, L., Chen, C., Tan, T., et al. (2022). Patients with breast cancer receiving chemotherapy: Effects of multisensory stimulation training on cognitive impairment. *Clin J Oncol Nurs* 26**,** 71-77.

Lyu, Y. R., Lee, H. Y., Park, H. J., Kwon, O. J., Kim, A. R., Jung, I. C., et al. (2022). Electroacupuncture for cancer-related cognitive impairment: A clinical feasibility study. *Integr Cancer Ther* 21**,** 15347354221098983.

Miao, X., Wu, H., Liu, Y., Zhang, S., Li, C., and Hao, J. (2022). Clinical efficacy of acupuncture on neoadjuvant chemotherapy with capecitabine plus paclitaxel and radiotherapy in progressive gastric cancer. *Journal of Oncology* 2022**,** 6156585.

Nelson, A. R., Sweeney, M. D., Sagare, A. P., and Zlokovic, B. V. (2016). Neurovascular dysfunction and neurodegeneration in dementia and alzheimer's disease. *Biochimica et Biophysica Acta (BBA) - Molecular Basis of Disease* 1862**,** 887-900.

Rostock, M., Jaroslawski, K., Guethlin, C., Ludtke, R., Schroder, S., and Bartsch, H. H. (2013). Chemotherapy-induced peripheral neuropathy in cancer patients: A four-arm randomized trial on the effectiveness of electroacupuncture. *Evid Based Complement Alternat Med* 2013**,** 349653.

Sawada, N. O., Zago, M. M. F., Galvão, C. M., Cardozo, F. M. C., Zandonai, A. P., Okino, L., et al. (2010). The outcomes of visualization and acupuncture on the quality of life of adult cancer patients receiving chemotherapy. *Cancer Nursing* 33.

Shen, Q., Deng, D., Li, G., Ruan, J., Shao, X., Wang, P., et al. (2025). Electroacupuncture frequency for chemotherapy-induced neuropathy in breast cancer: A randomized controlled trial. *The Oncologist* 30**,** oyaf262.

Tong, T., Pei, C., Chen, J., Lv, Q., Zhang, F., and Cheng, Z. (2018). Efficacy of acupuncture therapy for chemotherapy-related cognitive impairment in breast cancer patients. *Med Sci Monit* 24**,** 2919-2927.

Zeng, Y., Cheng, A. S. K., Song, T., Sheng, X., Wang, S., Xie, J., et al. (2018). Effects of acupuncture on cancer-related cognitive impairment in chinese gynecological cancer patients: A pilot cohort study. *Integr Cancer Ther* 17**,** 737-746.

Zhang, Z. J., Man, S. C., Yam, L. L., Yiu, C. Y., Leung, R. C., Qin, Z. S., et al. (2020). Electroacupuncture trigeminal nerve stimulation plus body acupuncture for chemotherapy-induced cognitive impairment in breast cancer patients: An assessor-participant blinded, randomized controlled trial. *Brain Behav Immun* 88**,** 88-96.
