# Supplementary figures and images for: Chemotherapy-related cognitive impairment and non-pharmacological interventions targeting the nervous system: a systematic review
Source: Front Psychiatry. 2026 Jun 2;17:1789794. doi: 10.3389/fpsyt.2026.1789794 (PMC13269268; doi:10.3389/fpsyt.2026.1789794)

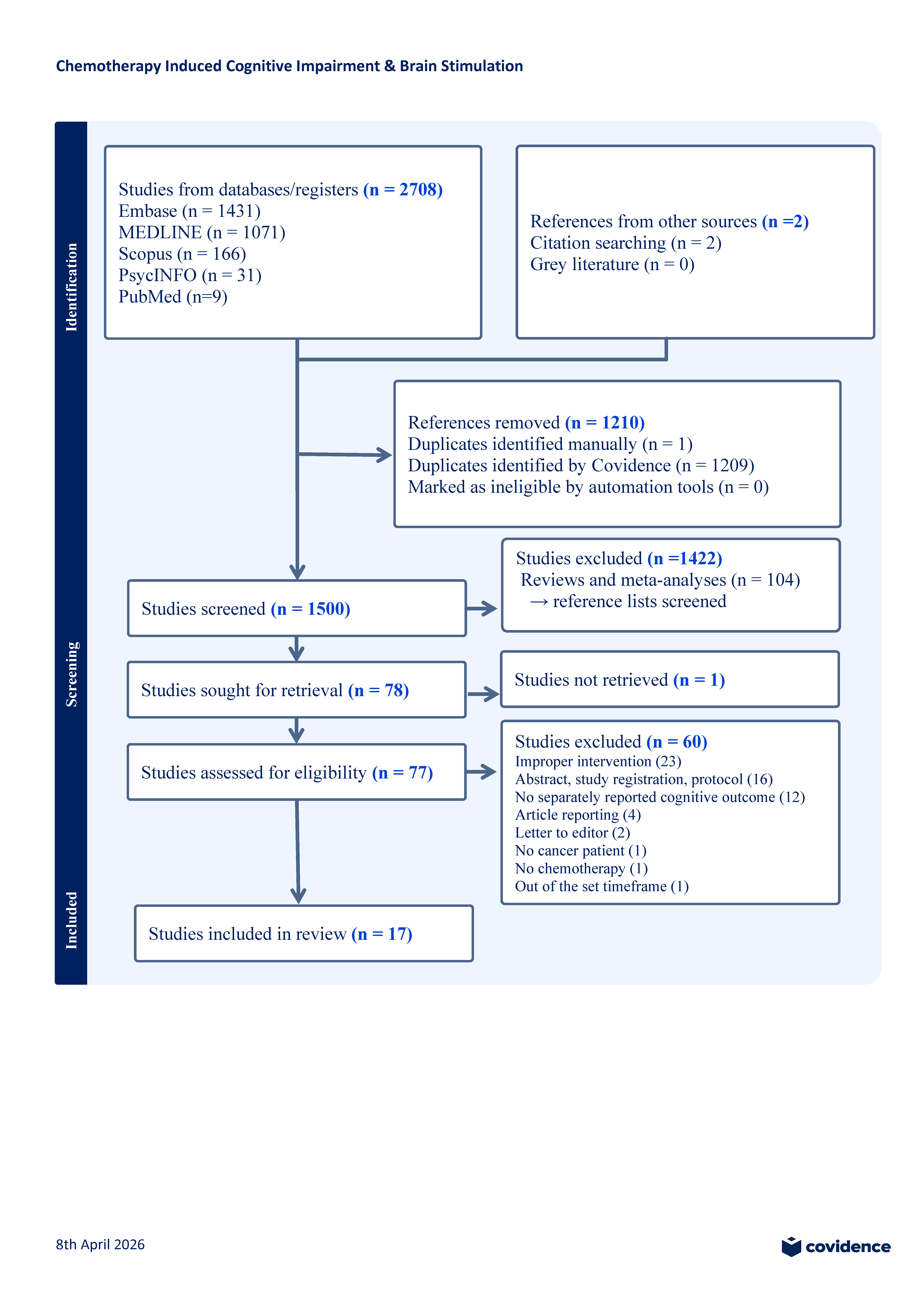

Supplement: Supplementary file 2 [file Image1.tif]
